# Functional gene assessment of bread wheat: breeding implications in Ningxia Province

**DOI:** 10.1186/s12870-021-02870-5

**Published:** 2021-02-18

**Authors:** Weijun Zhang, Junjie Zhao, Jinshang He, Ling Kang, Xiaoliang Wang, Fuguo Zhang, Chenyang Hao, Xiongfeng Ma, Dongsheng Chen

**Affiliations:** 1 Crop Research Institute, Ningxia Academy of Agriculture and Forestry Sciences, Yinchuan, 750002 Ningxia China; 2 State Key Laboratory of Cotton Biology, Institute of Cotton Research, Chinese Academy of Agricultural Sciences, Anyang, 455000 Henan China; 3 Key Laboratory of Crop Gene Resources and Germplasm Enhancement, Ministry of Agriculture and Rural Affairs/The National Key Facility for Crop Gene Resources and Genetic Improvement/Institute of Crop Sciences, Chinese Academy of Agricultural Sciences, Beijing, 100081 China

**Keywords:** Founder parents, KASP, Functional markers, Ningxia wheat

## Abstract

**Background:**

The overall genetic distribution and divergence of cloned genes among bread wheat varieties that have occurred during the breeding process over the past few decades in Ningxia Province, China, are poorly understood. Here, we report the genetic diversities of 44 important genes related to grain yield, quality, adaptation and resistance in 121 Ningxia and 86 introduced wheat cultivars and advanced lines.

**Results:**

The population structure indicated characteristics of genetic components of Ningxia wheat, including landraces of particular genetic resources, introduced varieties with rich genetic diversities and modern cultivars in different periods. Analysis of allele frequencies showed that the dwarfing alleles *Rht-B1b* at *Rht-B1* and *Rht-D1b* at *Rht-D1*, *1BL/1RS* translocation, *Hap-1* at *GW2-6B* and *Hap-H* at *Sus2-2B* are very frequently present in modern Ningxia cultivars and in introduced varieties from other regions but absent in landraces. This indicates that the introduced wheat germplasm with numerous beneficial genes is vital for broadening the genetic diversity of Ningxia wheat varieties. Large population differentiation between modern cultivars and landraces has occurred in adaptation genes. Founder parents carry excellent allele combinations of important genes, with a higher number of favorable alleles than modern cultivars. Gene flow analysis showed that six founder parents have greatly contributed to breeding improvement in Ningxia Province, particularly Zhou 8425B, for yield-related genes.

**Conclusions:**

Varieties introduced from other regions with rich genetic diversity and landraces with well-adapted genetic resources have been applied to improve modern cultivars. Founder parents, particularly Zhou 8425B, for yield-related genes have contributed greatly to wheat breeding improvement in Ningxia Province. These findings will greatly benefit bread wheat breeding in Ningxia Province as well as other areas with similar ecological environments.

**Supplementary Information:**

The online version contains supplementary material available at 10.1186/s12870-021-02870-5.

## Background

China is the largest wheat producer and consumer in the world, with an annual production area of approximately 2.7 Mha and a production yield of 133.6 Mt in 2019, accounting for 18% of wheat globally [1]. Bread wheat (*Triticum aestivum* L.) is widely distributed in intricate geographical environments in China, reflecting its wide adaptability and high yield. Ningxia, a northwestern province of China with complex ecological types, has a long agricultural history of wheat cultivation. Since the 1950s, bread wheat varieties have experienced five replacements: the wheat germplasm ‘Quality’ introduced from Australia was widely grown in the 1950s and achieved the first variety update of Ningxia spring wheat; the ‘Abbondanza’ wheat resource from Italy was efficiently used in the 1960s and achieved the second variety update of Ningxia wheat breeding; breeding of milestone variety ‘Doudi 1’ is representative of the third variety update in the 1970s; ‘Ningchun 4’ was one of the most used spring wheat varieties in China, and its application and improvement in the 1980s was the fourth variety update of Ningxia wheat; the release of ‘Ningchun 50’ in the 2000s was the mark of the last variety update [2]. ‘Abbondanza’, as a representation of founder parents, is widely planted (> 667,000 ha) in China [3]. Founder parents, which serve as important germplasm resources, are very important for the update of new varieties, and many modern wheat cultivars can be tracked to ancestral founder parents. Wheat germplasm introduction in Ningxia has played an important role in wheat breeding. Introduced bread wheat germplasms, such as ‘Quality’ and ‘Cajeme F-71’, have driven studies on wheat production areas with northward expansion and farming system reforming in the Yellow River Ningxia Basin, as well as wheat breeding programmes in Ningxia. Superior landraces in the early 1950s formed the basis for wheat improvement programmes and carried particular genetic resources for adaptation to local environmental conditions. Therefore, it is essential to dissect the genetic contributions to bread wheat improvement of landraces, introduced wheat varieties and specific founder parents at multiple gene levels in the past several decades to direct future wheat breeding in Ningxia.

Bread wheat has the characteristics of a large genome size, allopolyploid, highly complex repetitive genome contents shaped by two recent polyploidization events [4–7], domestication [8], gene flow from frequent intra- and inter-species introgression [9, 10], and post-domestication selection aimed at developing high-yielding locally adapted varieties [11]. Multiple factors drive the evolution of bread wheat varieties, particularly many important genetic loci that have been selected during modern wheat breeding. Insight into these genetic loci is important for understanding phenotypic variations in adaptability, resistance to biotic and abiotic stresses, processing and nutritional quality, and yield stability. The adaptation of wheat to diverse environments is largely governed by genes related to vernalization (*Vrn-A1*, *Vrn-B1* and *Vrn-D1*) [12], photoperiod (*Ppd-D1,* etc.) [13], and plant height (*Rht-B1* and *Rht-D1*) [14]. Yield-related genes include the sucrose synthase genes *TaSus1-7A*, −*7B* and *TaSus2-2A*, −*2B* for thousand-kernel weight and grain size [15, 16], *TaGW2-6A*, −*6B* for grain width [17–19], *TaGS-D1* for grain size [20], *TaCwi-A1* encoding cell wall invertase [21], *TaCKX6-D1* encoding cytokinin oxidase/dehydrogenase [22], and the grain length-associated gene *TaGASR-A1* [23]. Assessing processing quality is crucial in wheat quality improvement. Strong-gluten wheat varieties are characterized by a combination of medium-high kernel hardness, acceptable protein content, medium-strong dough and good extensibility; representative varieties include Yumai 34 and Zhengmai 366 for both pan bread and noodle quality [24]. High- and low-molecular-weight glutenin subunits (HMW-GS and LMW-GS) associated with dough quality are influenced by *Glu-1* and *Glu-3* loci [25, 26]. Flour colour responsible for noodle quality is influenced by several factors, including polyphenol oxidase (PPO) activity (*Ppo-A1* and *Ppo-D1*) [27, 28], phytoene synthase (PSY) enzymes (*Psy-A1*, *Psy-B1* and *Psy-D1*) [29, 30], ζ (zeta)-carotene desaturase (ZDS) enzymes (*Zds-A1*) [31] and peroxidase (*Pod-A1*) [32]. Kernel hardness, which has a profound effect on milling and end-use quality, is largely determined by the *Pina-D1* and *Pinb-D1* genes encoding puroindoline a and puroindoline b proteins, respectively [33]. Increasing biotic and abiotic stresses are major challenges that accompany the impacts of climate and environmental changes on wheat breeding. In recent decades, some important stress-resistance genes have been cloned. As one of the response drought factors, dehydration-responsive element-binding (DREB) proteins encoded by the *Dreb-B1* locus are induced to improve drought tolerance [34]. Fusarium head blight (FHB) devastates wheat production worldwide, and its resistance genes *Fhb1* using recombinants [35, 36] and *Fhb7* in wheat distant hybridization breeding [37] were cloned recently. The *Lr34/Yr18/Pm38* locus conferring durable adult plant resistance to multiple diseases is used in wheat breeding programmes worldwide [33]. The 1BL/1RS translocation (*1BL/1RS*) has been widely adopted in wheat breeding due to its positive impacts on grain yield, adaptation, and, in particular, the presence of resistance genes to several diseases and pests, though the translocation is associated with undesirable bread-making quality [38].

Modern breeding has imposed selection for improved productivity that largely influences the frequency of superior alleles for genetic loci underlying traits of breeding interest. Therefore, molecular diagnosis of allelic variations is important to manipulate beneficial alleles in molecular breeding of wheat. Enhanced sequencing capacity, along with the availability of high-quality genome sequences of bread wheat, has allowed researchers to identify specific favorable alleles using molecular markers. Currently, 157 functional markers documented for more than 100 loci related to adaptability, resistance to biotic and abiotic stresses, quality and grain yield have been converted into high-throughput KASP assays [39]. Such approaches will promote assessing the distribution of functional genes of wheat germplasms and applications in bread wheat breeding.

Our objectives for this study were to evaluate the genetic structure, diversity, divergence and allelic variations of bread wheat germplasm resources in Ningxia Province, China, using KASP assays of 44 cloned genes for adaptation, stress resistance, quality, and grain yield. Genetic characteristics were evaluated in 207 bread wheat cultivars, landraces and advanced lines, including founder parents and varieties from Ningxia and other regions. Gene flow and allelic frequency implicate the distribution of important functional genes, which may improve the selection of future wheat breeding in Ningxia Province and provide a robust breeding foundation to be used as a guide for other regions and countries with similar ecological environments.

## Methods

### Plant materials and DNA extraction

A representative sampling of bread wheat germplasm consisting of 207 wheat varieties, including 121 Ningxia varieties and 86 introduced varieties, was evaluated (Table S1). The latter were introduced to Ningxia Province over past decades and played a huge role in local wheat breeding. The Ningxia varieties included 13 landraces and 108 modern cultivars and advanced lines. In addition, six founder parents among the 207 wheat varieties used in this study included Moba 66, Abbondanza, Beijing 8, Orofen, Xiaoyan 6 and Zhou 8425B. Genomic DNA was extracted from fresh leaves from each accession using the CTAB method [40].

### KASP genotyping of functional genes

Conventional functional markers were summarized based on 44 cloned wheat genes for grain yield, quality, adaptation and stress resistance [33]; these markers were converted into KASP assays [39] that have been widely exploited to characterize wheat germplasm resources [41–44]. A total of 44 KASP arrays developed from cloned genes were used for genotyping in this study (Table S2). The KASP arrays were designed based on diagnostic SNP markers following standard KASP guidelines. Primers were designed carrying a standard FAM tail (5′-GAAGGTGACCAAGTTCATGCT-3′) and HEX tail (5′-GAAGGTCGGAGTCAACGGATT-3′) with different fluorescence signals.

KASP assays were performed in 5.0 μL mixtures containing 2.2 μL of 40 ng/μL DNA, 2.5 μL of 1xKASP V4.0 2X Master mix (KBS-1016-017), 0.04 μL Mg^2+^, 0.056 μL of primer mixture, and 0.204 μL ddH_2_O and the following amplification programme: denaturation at 95 °C for 15 min, followed by ten touchdown cycles (95 °C for 20 s; touchdown at 65 °C initially and decreasing by 1 °C per cycle for 25 s) and 30 additional cycles of annealing (95 °C for 10 s; 57 °C for 60 s) [44]. KASP genotyping was performed using QuantStudio™ 7 Flex (Applied Biosystems by Life Technologies, U.S.). Each sample carrying different fluorescence signals was visualized, and the corresponding data was generated with QuantStudioTM Real-time PCR Software v1.3 (Applied Biosystems by Life Technologies) (Fig. S1). Then, we manually converted these data to allelic varieties according to the corresponding fluorescence tails.

### Population structure and phylogenetic analysis

A neighbour-joining tree was constructed in PowerMarker v3.25 [45] and visualized in MEGA 7 [46] using genotypic data for 44 genes. The first three eigenvectors of principal coordinate analysis (PCA) were obtained using the R package Adegenet v2.0.1 [47]. The population structure of the 207 accessions based on the 44 functional genes was evaluated using Structure 2.3.4 with a burn-in period at 50,000 iterations and a run of 500,000 replications of Markov Chain Monte Carlo (MCMC) [48]. The number of populations was estimated based on the ΔK model [49].

Allele numbers and frequencies were calculated for all loci. Genetic diversities were evaluated by PowerMarker v3.25, and Student’s *t*-test was applied to compare the effects of two genotypes at a threshold probability of *P* < 0.05. Genetic flow and *F*-statistics (*Fst*) were measured for population differentiation with POPGENE software [50].

## Results

### Genotyping and population structure

Genotyping of 207 bread wheat varieties using 44 KASP assays identified allelic variations at 44 loci (Table S1). All selected KASP assays exhibited clear clustering results for the varieties (Fig. S1). In total, these loci are related to grain yield (10), quality (14), adaptation (6), and stress resistance (14).

**Fig. 1:**
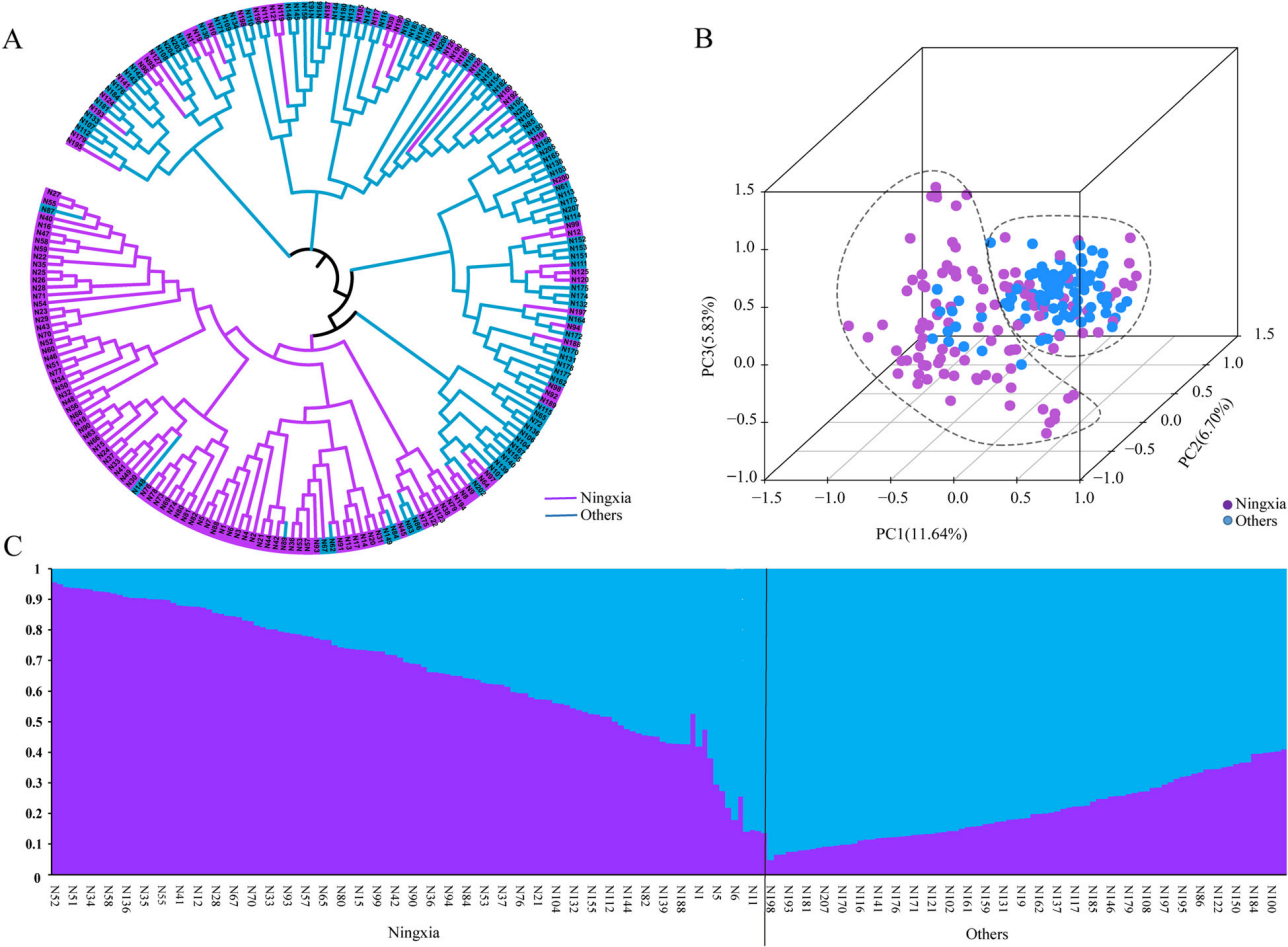
The population structure of 207 wheat accessions based on 44 genes. **a** A neighbour-joining tree of all accessions. Different lines are presented in different colors. **b** Plots of first three principal components of all accessions. **c** Population structure of all accessions based on Structure

The neighbour-joining analysis divided 207 varieties into two groups, namely, Ningxia and Others (Fig. 1a), in agreement with PCA (Fig. 1b). The number of subpopulations (K) was plotted against the ΔK calculated from the structure, and the peak of the broken line graph was observed at K = 2 (Figs. 1c, S2), demonstrating that the population was basically divided into two subgroups. The first subgroup mainly referred to landraces and cultivars from Ningxia Province (Ningxia); the second mainly consisted of introduced varieties from foreign countries and other provinces in China (Others). Moreover, accessions from Ningxia grouped into two clades of landraces and modern cultivars (Fig. S3). This indicated the characteristics of the genetic components of Ningxia wheat, in which landraces, introduced varieties and modern cultivars in different periods together formed wheat breeding processes.

### Significant genetic divergence in yield and quality genes between accessions from Ningxia and others

**Fig. 2:**
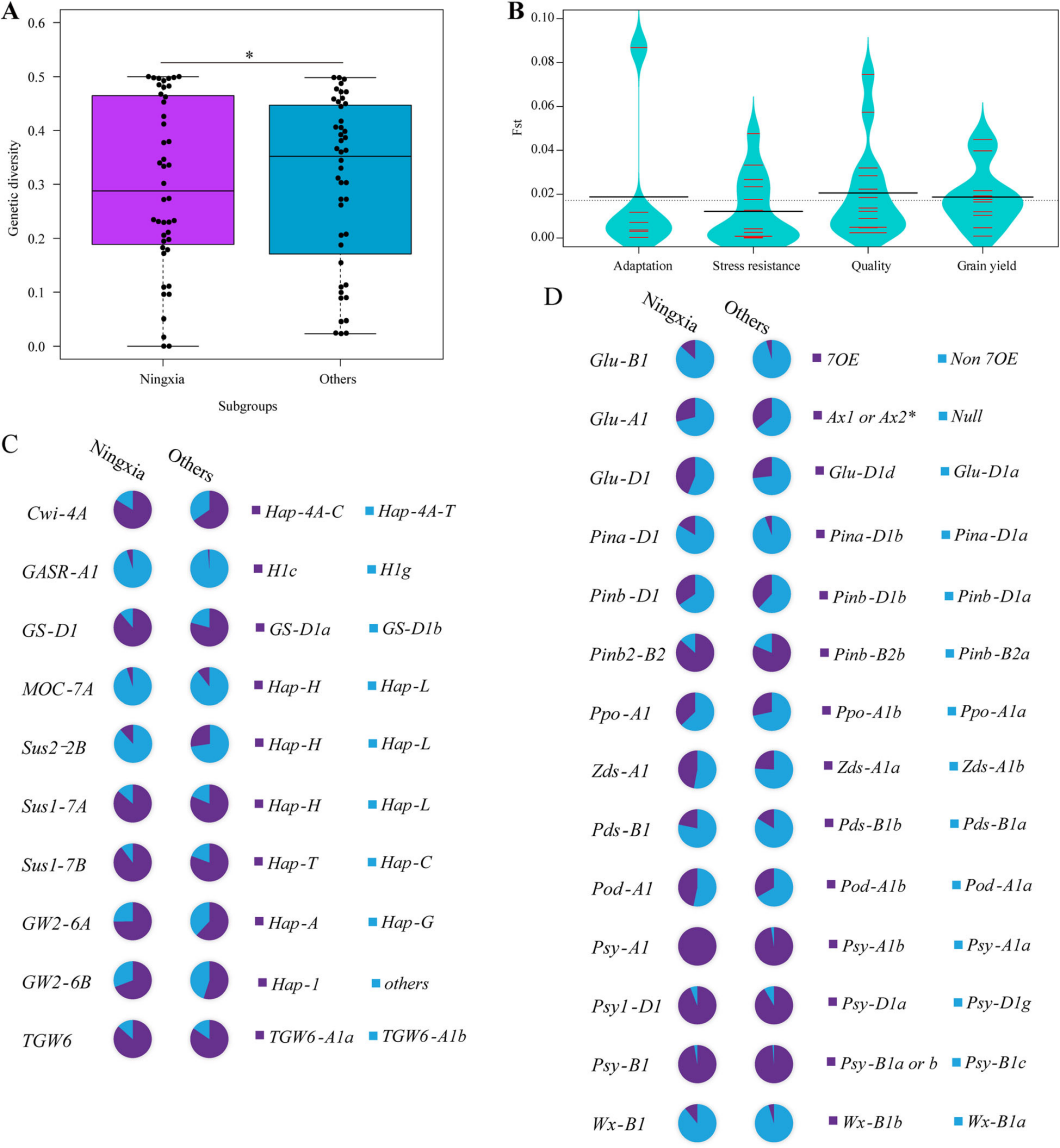
Genetic divergence on all 44 genes between Ningxia and Others subgroups. **a** Genetic diversities between Ningxia and Others subgroups. **b** Genetic differentiation (*Fst*) between Genetic diversities on grain yield genes. **c** Allele frequency at grain yield genes between Ningxia and Others subgroups. **d** Allele frequencies of quality genes between Ningxia and Others subgroups

Genetic diversity and variations were assessed to further clarify the large genetic differences between germplasms from Ningxia and Others. There was apparent difference in genetic diversity at 44 loci controlling yield, quality, adaptation and stress resistance between Ningxia wheat germplasms and Others (Fig. 2a). Further exploration indicated a higher genetic diversity at ten grain yield loci in Others than in the Ningxia wheat varieties (*P* < 0.01), whereas Ningxia showed higher genetic diversity than Others at 14 quality genes (*P* < 0.05) (Fig. S4A, B). Among them, estimated genetic diversity at the *Cwi-4A*, *GS-D1*, *Sus2-2B* and *Sus1-7B* loci for yield was abundant in the Others subgroup, whereas *Glu-B1*, *Glu-D1*, *Pina-D1* and *Zds-A1*, which are related to quality, showed much higher genetic diversity in the Ningxia subgroup than in the Others subgroup (Table S3). In addition, we found that genetic divergence was most obvious for quality genes, followed by yield genes (Fig. 2b). In-depth analysis revealed evident genetic divergence at some loci, such as *Cwi-4A* (0.035) and *Sus2-2B* (0.035) for grain yield, *Pinb-D1* (0.057) and *Zds-A1* (0.064) for quality in the Ningxia and Others subgroups (Table S3).

Allele frequency analysis showed that alleles of *Hap-4A-C* (*Cwi-4A*), *GS-D1a* (*GS-D1*), *Hap-A* (*GW2-6A*) and *Hap-1* (*GW2-6B*) for larger grain size and TKW were predominant in the Ningxia subgroup compared with the Others subgroup (Fig. 2c), whereas at *Sus2-2B*, the allele *Hap-H* associated with higher TKW was more frequent in the Others subgroup. At 14 loci for quality traits, a higher frequency of *Glu-D1d* encoding the high-molecular-weight glutenin subunit (HMW-GS) Dx5 + Dy10 occurred more frequently in Ningxia (44%) than in Others (27%) (Fig. 2d). The *Ppo-A1b*, *Pod-A1b* and *Zds-A1a* alleles, associated with lower PPO activity, higher POD activity and lower yellow pigment content, respectively, were more frequent in Ningxia than in Others. In contrast, the frequencies of HMW-GS Ax1 or Ax2* and *Pinb-D1b* for hard grain texture were significantly higher in Others. Concerning adaptation and stress resistance genes, genetic diversity in the two subgroups was not significantly different (Fig. S4C, D), and allelic variations showed minor differences at *Rht-B1*, *Rht-D1*, *Vrn-B1*, *Vrn-D1* and *Ppd-D1* in both subgroups (Ningxia and Others) (Fig. S5).

### Genetic divergence in adaptation genes was most significant during breeding improvement in Ningxia Province

**Fig. 3:**
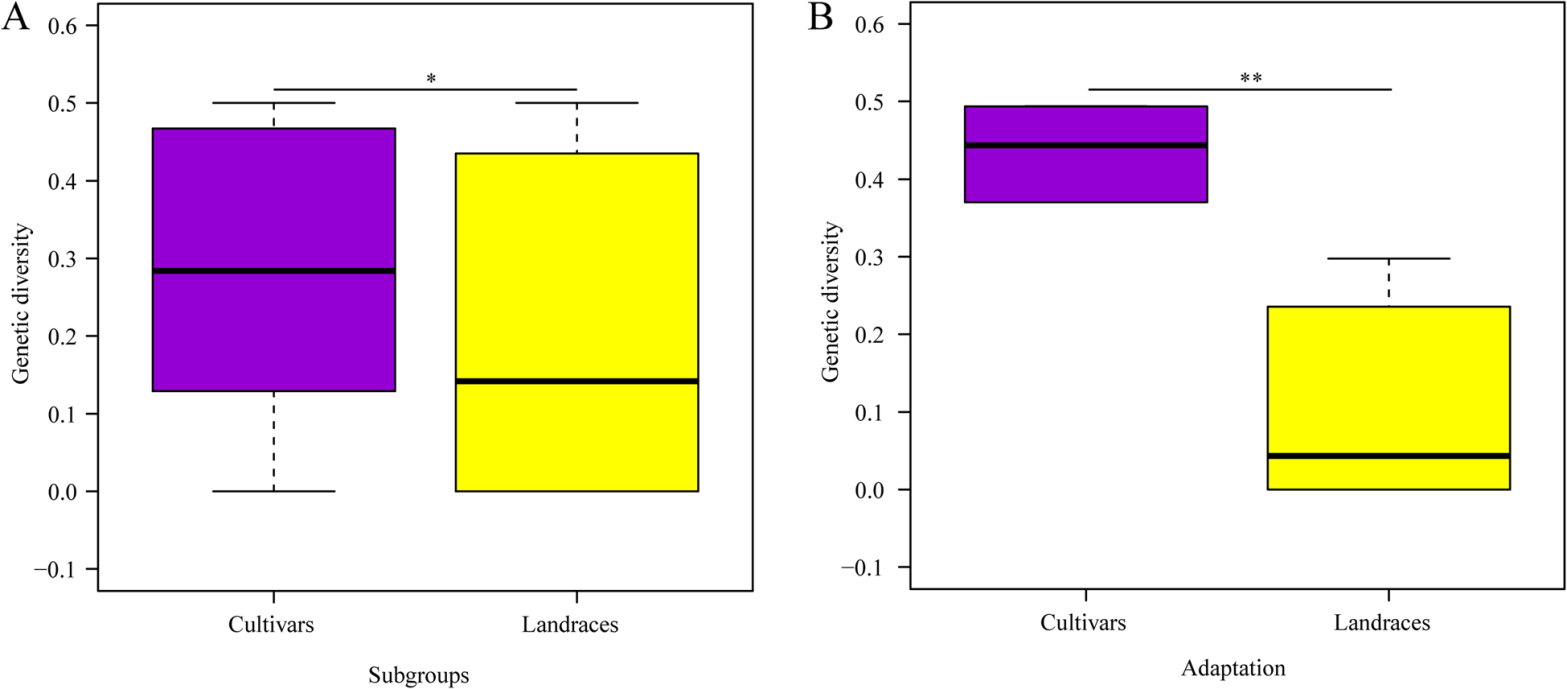
Genetic diversity between modern cultivars and landraces in Ningxia Province. **a** Genetic diversity based on 44 genes. **b** Genetic diversities on adaptation genes

**Fig. 4:**
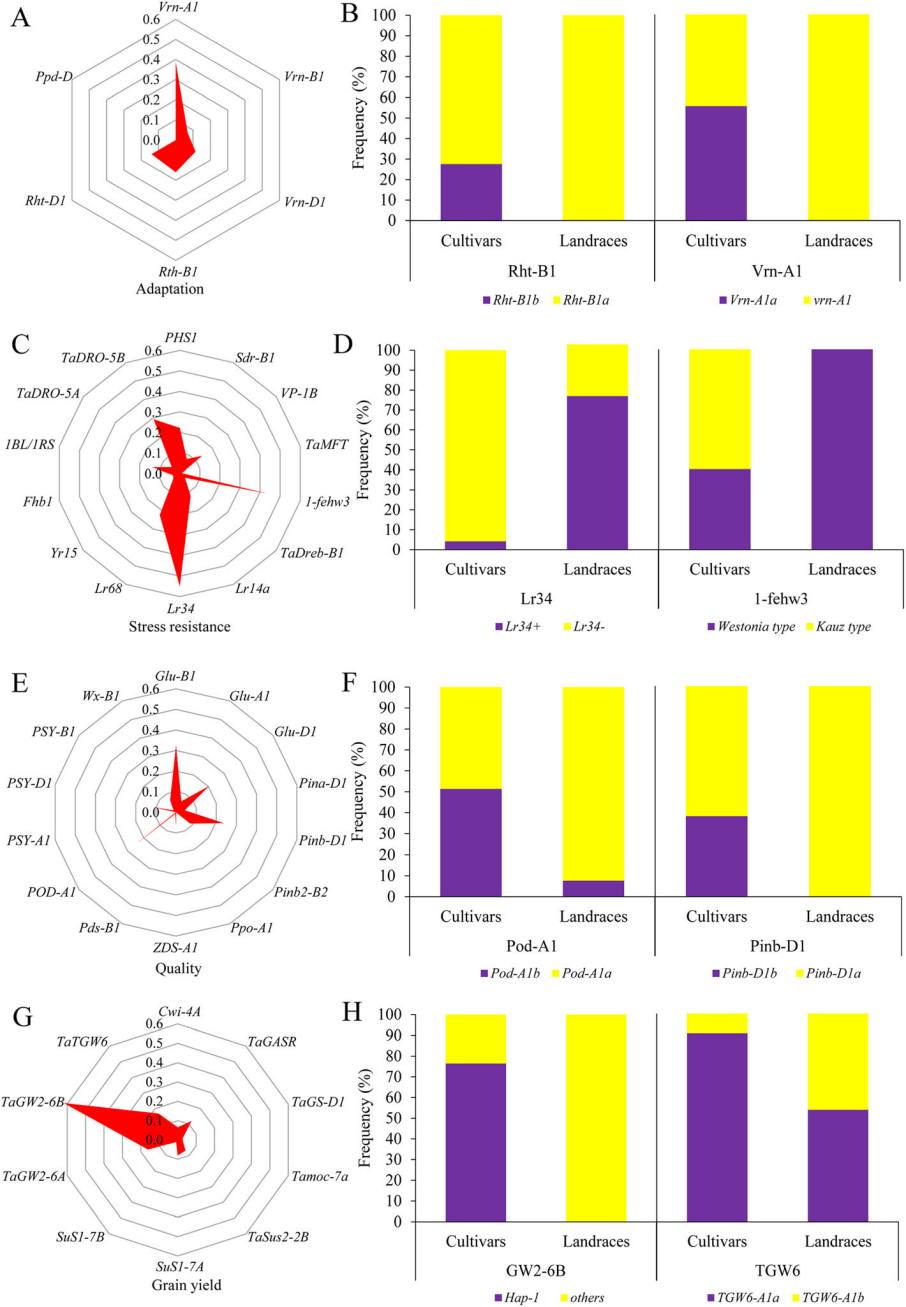
Genetic differentiation (*Fst*) and allele frequencies of 44 genes between modern cultivars and landraces in Ningxia Province. **a**, **b** *Fst* and allele frequencies at adaptation genes between modern cultivars and landraces. **c**, **d** Fst and allele frequencies of stress resistance genes between modern cultivars and landraces. **e**, **f** *Fst* and allele frequencies of quality genes between modern cultivars and landraces. **g**, **h** *Fst* and allele frequency of grain yield-related genes between modern cultivars and landraces

To evaluate population differentiation during breeding improvement in Ningxia Province, we further analysed the genetic relationships between landraces and modern cultivars. Higher genetic diversity occurred in modern cultivars than in landraces (Fig. 3a). Moreover, the difference in genetic diversity was clear in adaptation-related genes (Fig. 3b). Population differentiation (*Fst*) between modern cultivars and landraces was very high at *Vrn-A1* (0.39), followed by *Rht-B1* (0.16) (Fig. 4a). Similarly, the spring-type allele *Vrn-A1a* at *Vrn-A1*, which influences vernalization, was frequently found in modern cultivars (57%) but was not detected in landraces (Fig. 4b). In contrast, *Vrn-B1b*, which is associated with the spring type, is predominant in both modern cultivars (58%) and landraces (82%), and *Vrn-D1a,* also related to the spring type, retained towering scaling in modern cultivars (57%) and landraces (86%) (Fig. S7). The dwarfing allele *Rht-B1b* (*Rht-B1*) is present in 28% of modern cultivars but is absent in landraces (Fig. 4b). A similar situation is observed for another dwarfing gene, *Rht-D1b*. Interestingly, the photoperiod-insensitive allele (*Ppd-D1a*) predominates in modern cultivars and landraces (Fig. S7).

Population differentiation in other genes controlling yield, quality and resistance was also analysed, though no significant differences were found between the two groups (Fig. S6). For genes controlling stress resistance, the *Fst* values among the two subgroups at the loci *Lr34* and *1-fehw3* were extremely high, at 0.55 (*Lr34*) and 0.43 (*1-fehw3*) respectively, compared with those of other resistance genes (Fig. 4c). Allele frequency analyses showed the favorable alleles *Lr34+* and *Westonia type* at *1-fehw3* to be predominant in landraces (Fig. 4d). The genetic differentiation (*Fst*) values for *DRO-5B*, *Lr68*, *PHS1*, *VP-1B* and *Lr14a* were 0.30, 0.22, 0.22, 0.14 and 0.12, respectively (Table S4), whereas corresponding favorable allele frequencies showed distinct differences between modern cultivars and landraces (5% vs 54, 5% vs 47, 50% vs 92, 67% vs 30, 22% vs 0%) (Fig. S8). For quality genes, the most extreme genetic differentiation between the two subgroups was detected for *Pod-A1* (0.23), followed by *Pinb-D1* (0.24) (Fig. 4e). The majority of modern cultivars (55%) carry the *Pod-A1b* allele, whereas few landraces harbour this allele. The hard grain texture allele (*Pinb-D1b*) is frequently present in modern cultivars (38%), but it is absent in landraces (Fig. 4f), as verified by genetic differentiation between the two subgroups (Fig. 4e). Additionally, loci including *Glu-D1*, *PSY1-D1* and *Pinb2-B2* show obvious genetic differences (Table S4), and the corresponding allele frequencies differ significantly between cultivars and landraces (Fig. S9). For yield-related genes, the most significant difference occurred in *TaGW2-6B* (Fig. 4g), at which the favorable allele *Hap-1* predominates in modern cultivars (76%) but is absent in landraces (Fig. 4h). At the *TGW6*, *Cwi-4A* and *GS-D1* loci, favorable allele frequencies are higher in modern cultivars than in landraces (Fig. S10), whereas the opposite situation is observed at the loci *GASR-A1*, *Sus1-7A* and *GW2-6A*.

### Genetic contribution from founder parents for Ningxia bread wheat cultivars

**Fig. 5:**
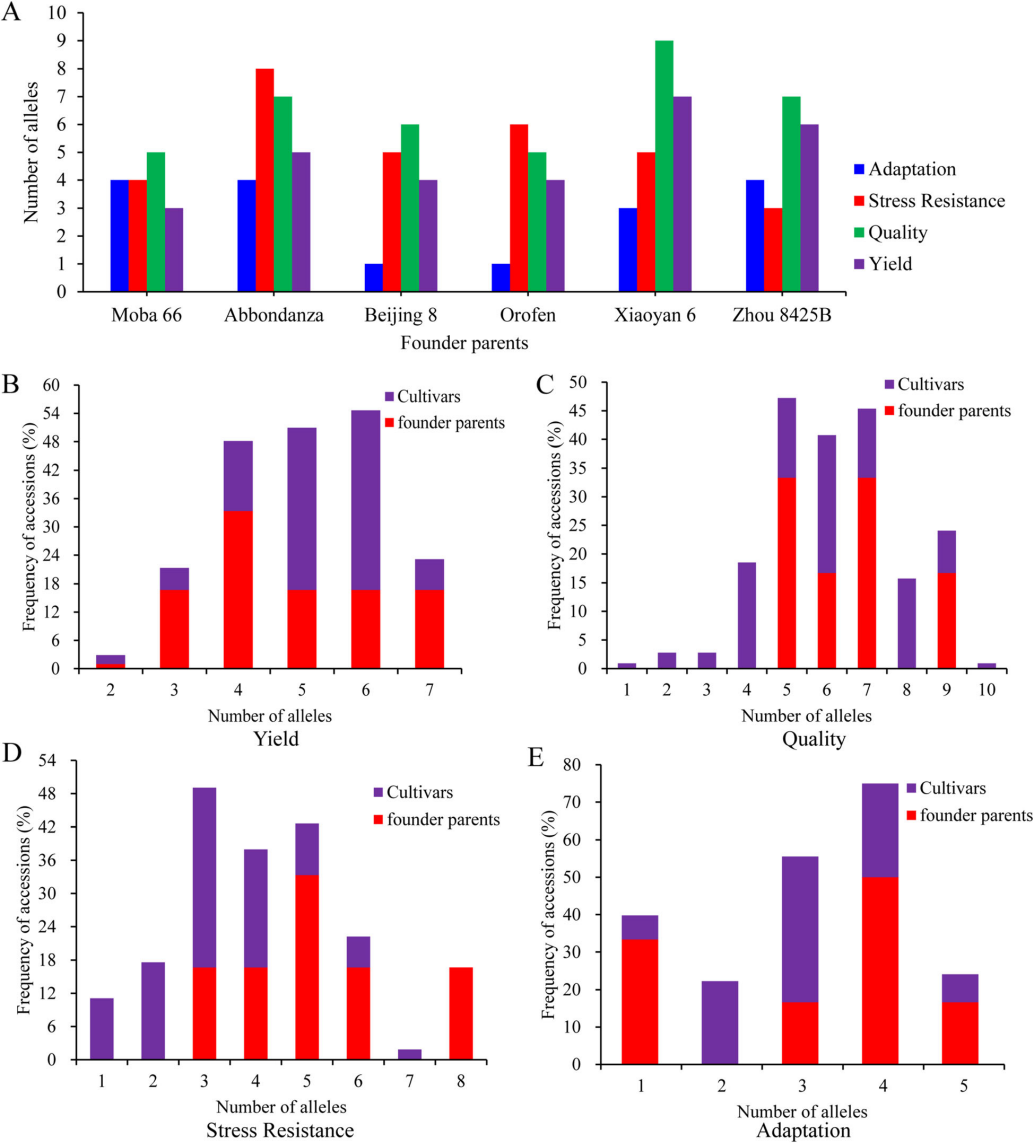
Distributions of favorable alleles of yield, quality, stress resistance and adaptation genes in founder parents and modern cultivars. **a** Number of favorable alleles at four types of genes in six founder parents. **b** Proportion of accessions carrying different numbers of favorable alleles of yield genes in founder parents and modern cultivars. **c** Proportion of accessions carrying different numbers of favorable alleles of quality genes in founder parents and modern cultivars. **d** Proportion of accessions carrying different numbers of favorable alleles of stress resistance genes in founder parents and modern cultivars. **e** Proportion of accessions carrying different numbers of favorable alleles of adaptation genes in founder parents and modern cultivars

Founder parents, as an important genetic resource, have greatly promoted the improvement of wheat varieties in China since the 1950s. In this study, we analysed the genetic contributions of six founder parents, including Moba 66, Abbondanza, Beijing 8, Orofen, Xiaoyan 6 and Zhou 8425B, to modern cultivars in Ningxia Province. To clearly understand the importance of founder parents, we counted the number of favorable alleles of genes for yield, quality, adaptation and stress resistance in these cultivars (Fig. 5). The number of favorable alleles for higher TKW in the six founder parents ranged from three to seven for ten yield genes. The founder parent Xiaoyan 6 carries seven favorable alleles: *Hap-4A-C* at *Cwi-4A*, *GS-D1a* at *GS-D1*, *Hap-H* at *Sus1-7A* and *2B, Hap-A* at *GW2-6A, Hap-1* at *GW2-6B* and *TGW6-A1a* at *TGW6* (Fig. S11). For 14 quality genes, the average number of favorable alleles was six, ranging from five to nine. The founder parent Xiaoyan 6 carries the most favorable alleles: Ax1 or Ax2* at *Glu-A1*, *Glu-D1d at Glu-D1*, *Zds-A1a* at *Zds-A1*, *Pds-B1b* at *Pds-B1*, *Pod-A1b* at *Pod-A1*, *Psy-A1b* at *Psy-A1, Psy-D1a* at *Psy1-D1*, *Psy-B1a or b* at *Psy-B1* and *Wx-B1b* at *Wx-B1*. All six founder parents carry *Psy-A1b*, *Psy-D1a* and *Psy-B1a or b* associated with low YP content, except *Psy-D1* for Zhou 8425B (Fig. S12). For stress resistance, the founder parent Abbondanza harbours eight favorable alleles, including *PHS+* at *PHS1, Vp-1Bc* at *VP-1B, PHS+* at *MFT-A1, Westonia type* at *1-fehw3, Dreb-B1a* at *Dreb-B1, Hap-5A-A* at *DRO-5A, Hap-5B-II* at *DRO-5B* and *Lr14+* at *Lr14a* (Fig. S13). For six adaptation genes, the photoperiod-insensitive allele (*Ppd-D1a*) was detected in all founder parents, except for Orofen. In addition, Moba 66, Xiaoyan 6 and Zhou 8425B have the dwarfing allele *Rht-B1b*. Zhou 8425B also has another dwarfing allele, *Rht-D1b* (Fig. S14). Such evaluation of these founder parents with respect to different types of functional genes allowed us to infer contributions to breeding improvement in Ningxia Province.

To compare differences in four types of genes between founder parents and modern cultivars, we investigated the relationship number of alleles with the proportion of accessions. Seventeen percent of the founder parents had seven and nine favorable alleles at yield and quality genes, respectively; the proportion was 7% on average for modern cultivars (Figs. 5b, c). Most founder parents and modern cultivars have three to six allelic variations in resistance genes and carry dwarfing, spring-type and photoperiod-insensitive alleles at adaptation genes (Figs. 5d, e).

**Fig. 6:**
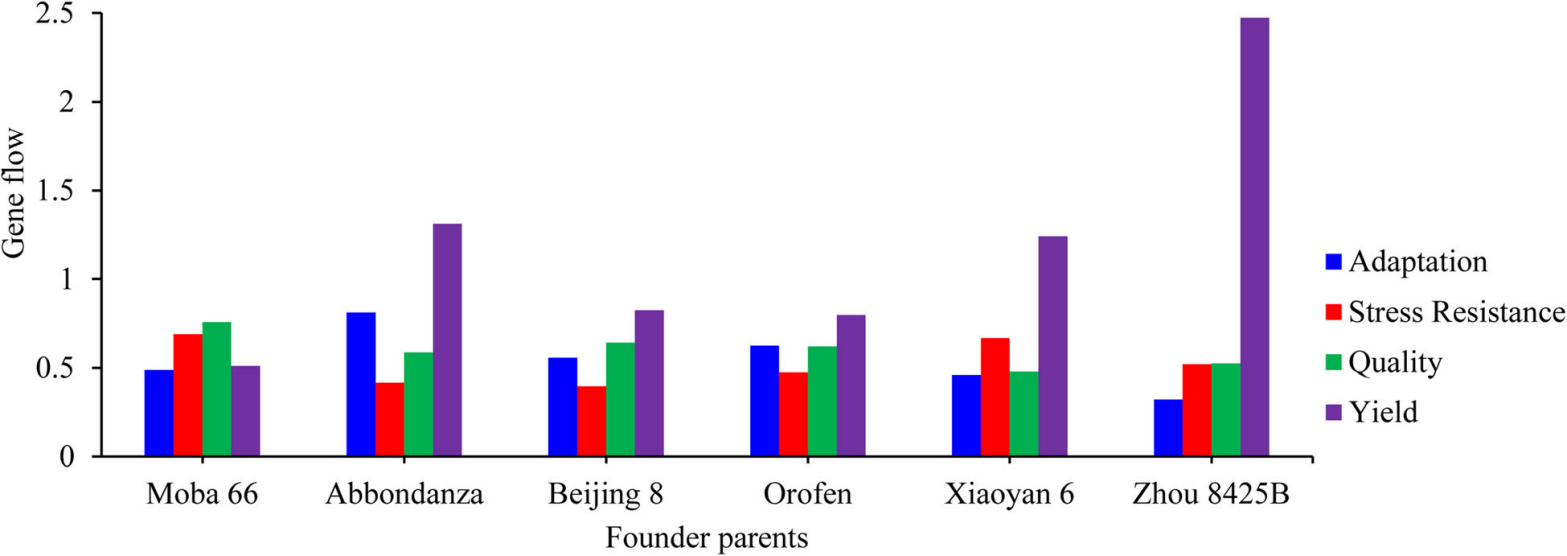
Gene flow between six founder parents and modern cultivars at yield, quality, stress resistance and adaptation gene

The gene flow value at yield genes was 2.47 between modern cultivars and Zhou 8425B, which was the most frequent among all founder parents, indicating that Zhou 8425B had the largest genetic exchange with modern cultivars and played an important role regarding yield potential in Ningxia wheat (Fig. 6). At quality and resistance genes, all founder parents had nearly equal gene flow to modern cultivars, with an average gene flow of 0.60 at quality loci ranging from 0.48 to 0.76 and 0.53 at resistance loci ranging from 0.40 to 0.69. For adaptation genes, the gene flow values between the founder parents Abbondanza, Orofen and modern cultivars were 0.82 and 0.63, respectively. In summary, founder parents with different favorable alleles together were responsible for improvement of Ningxia wheat cultivars.

## Discussion

### Population structure indicating wheat genetic components in Ningxia Province

Ningxia Province, which has diverse ecological types, is not only suitable for the growth of winter wheat but also the main cultivation area for spring wheat. The practice of introducing foreign germplasm resources shows that almost all types of bread wheat can be planted and harvested normally in Ningxia Province. Of course, landraces, as local characteristic resources, have played an important role in early breeding programmes. In this study, all varieties were clustered into three subgroups based on population structure analyses, namely, modern cultivars, landraces and Others, revealing their genetic differences based on 44 important functional genes (Figs. S3). To further clarify the breeding contribution of introduced germplasm and landraces in Ningxia Province, genetic diversity and the frequency spectrum for divergence were evaluated based on 44 genes for yield, quality, adaptation and resistance. In this study, genetic diversity was most enriched for the subgroup Others comprising introductions outside of Ningxia Province, followed by modern cultivars; landraces showed minimum genetic diversity at 44 important loci (Figs. 2a, 3a). This indicates that conventional artificial hybridization breeding using external resources to improve varieties has increased diversity by promoting gene exchange and recombination in coding regions, particularly for important cloned functional genes for self-pollinated wheat [51]. Introduced varieties from other regions with rich genetic diversity have facilitated local breeding improvement; thus, introduced varieties have made a significant genetic contribution to Chinese modern cultivars [44]. The allele frequency spectrum for divergence also supports this at the gene level. Dwarfing alleles *Rht-B1b* at *Rht-B1* and *Rht-D1b* at *Rht-D1*, well-known Green Revolution genes that swept through China and had a significantly positive influence on wheat breeding, are very widespread in modern Ningxia cultivars and varieties introduced from other regions but absent in landraces (Figs. S5A, S7), indicating that introduced wheat germplasm with numerous beneficial genes is vital for broadening the genetic diversity of Ningxia wheat cultivars. Similar types of genes also include the *1BL/1RS* translocation, *GW2-6B*, and *Sus2-2B* (Figs. 2c, S8, S10). Therefore, wide introduction, in-depth research and effective use of wheat germplasm resources outside of Ningxia Province are important ways to improve wheat yield and breeding effectiveness in Ningxia Province. As autochthonous traditional varieties, evaluation of wheat landraces stored in gene banks with highly beneficial untapped diversity and sources of stress adaptation should be used for wheat improvement [52]. Due to the colonization of diverse ecological environments in the process of domestication and selection by ancient farmers in Ningxia Province, landraces contain broader specific genetic loci than most breeding programmes and form the basis of early wheat breeding, especially for China in the pre-1950s. Zhou et al. [53] highlighted environmental stresses and independent selection efforts that have resulted in considerable genome-wide divergence at the population level in Chinese wheat landraces. Of course, this characteristic has been exploited in other countries, where the first improved varieties consisted of selections of local landraces [52], such as the landrace population ‘Catalan de Monte’ in Spain [54, 55] and ‘Turkey Red’ in the United States [56]. Therefore, finding new genes and increasing the frequency of rare alleles among landraces in Ningxia Province via next-generation genotyping and sequencing technologies should be used in breeding. Overall, our results indicate that landraces with good adaptation and introduced varieties with wide diversities will co-promote bread wheat breeding in Ningxia Province.

### Dissecting allele frequency identifies the direction of important gene selection

Modern wheat breeding practices accompanying intensive selection pressure have always focused on economically important loci [57, 58]. For each of those loci contributing to agronomic phenotypes, causal polymorphisms have been identified with increased frequencies of favorable alleles consistent with selection during modern breeding [59]. In this study, we found evidence of convergent increases in allele frequencies at targeted genes for improved selection for Ningxia bread wheat from landraces to modern cultivars. *VP-1B* is one of important seed dormancy genes for PHS tolerance during harvest [60], and the favorable allele *Vp-1Bc* was found to be predominant, with a frequency of 68% in Ningxia modern cultivars. Similarly, *Hap-1* at the *GW2-6B* locus strongly influences kernel width and thousand-kernel weight, and the allele was found in 76% of modern cultivars, while the desirable allele was absent in landraces, indicating that breeders have intensively selected for the favorable allele at the *GW2-6B* locus due to the demand for increasing grain yield in breeding (Fig. 4h). HMW-GS is influenced at *Glu-D1* locus [25]. The allele *Glu-D1d* associated with a high gluten content and superior bread-making quality showed a relatively high frequency in modern cultivars compared with landraces, which is in agreement with reports for most cultivars in Pakistani and China [22, 57, 58]. In addition, the favorable allele *Hap-4A-C* at *Cwi-4A*, which encodes the CWI enzyme that converts sucrose to glucose associated with grain size, was detected in 85% of modern cultivars, showing the effective use of this gene in bread wheat molecular breeding.

For some important genes, their favorable alleles had maintained high values in varieties before modern wheat breeding [44]. The photoperiod-insensitive allele *Ppd-D1a* was found to be fixed at a frequency of 100% in both landraces and modern cultivars, showing that this gene is so important that it has been selected for completely before modern breeding in Ningxia. Flowering time is one of the most important developmental traits for wheat adaptability and yield stability in target environments, and the photoperiod-insensitive allele of the photoperiod response gene *Ppd1* is known to be a major determinant of flowering time optimization [61]. Therefore, early flowering in varieties carrying photoperiod-insensitive alleles was fixed during the long-term selection process. A high yellow pigment content is favored for durum wheat pasta but is considered undesirable for Chinese steamed bread and white noodles [62–64], and the alleles *Psy-A1b* and *Psy-B1a/b* are thus encouraged. The frequencies of the two alleles approached almost 100%; these alleles were fixed in both landraces and modern cultivars before breeding selection in Ningxia.

However, to breed perfect bread wheat varieties in Ningxia, favorable alleles with minor frequencies in modern cultivars should be regarded. For example, the root architecture-related gene *DRO-5B* is an IAA-response gene that is responsible for reduced height and increased thousand-kernel weight [65, 66]. The favorable allele *Hap-5B-II* is present in 54% of landraces but only 5% of modern cultivars, indicating that this allele might have undergone negative selection and thus underutilization in modern breeding. In addition, *H1c* at the *GASR-A1* locus, which influences grain length, is predominant in landraces but has a low frequency in modern cultivars. Overall, breeding is a process of aggregating desirable genes and eliminating undesirable or even deleterious alleles. Low frequencies of favorable alleles for important genes in modern cultivars identify the direction of improvement for future bread wheat breeding in Ningxia and are helpful for further breeding by design.

### Founder parents contain a combination of important functional genes

Founder parents, which serve as important germplasm resources, play a pivotal role in updating new varieties [67]. They exhibit not only superior phenotypes and high recombination ability but also wide adaptation and prominent specific characteristics [3]. Previous studies have found that genes controlling important traits were present in combination rather than being randomly distributed on chromosomes in founder parents [68–72]. For example, pedigree analysis of Huanghuazhan rice showed that 61.79% of 50 kb blocks are HTBs (Huanghuazhan traceable blocks), together with the elite performance of Huanghuazhan, and that large-scale important genes are located in HTBs, supporting that they represent the combination of elite alleles of important genes [73]. In our study, counting the number of favorable alleles at ten yield-related genes successfully clarified 3 ~ 7 favorable alleles for higher TKW in six founder parents. Furthermore, *Hap-4A-C* at *Cwi-4A*, *Hap-H* at *Sus1-7A*, *GS-D1a* at *GS-D1*, *Hap-A* at *GW2-6A*, *Hap-1* at *GW2-6B* and *TGW6-A1a* at *TGW6* are conserved in the founder parents Zhou 8425B, Xiaoyan 6 and Abbondanza, except for *GS-D1a* allele. For quality-related genes, the average number of favorable alleles was found to be approximately six, and *Psy-A1b*, *Psy-D1a*, *Psy-B1a* or *-b* associated with a low YP content are conserved across all founder parents, showing that favorable alleles of these important genes are conserved in modern bread wheat breeding. Interestingly, the six founder parents carry different favorable alleles at resistance- and adaptation-related genes, probably because these genes are randomly selected to respond to various environments, such that these founder parents can maintain high yield and good quality wherever they are cultivated.

Founder parents have excellent allele combinations of important genes for agronomical desirable traits, and many varieties have been derived from them. In this study, yield trait improvement of modern cultivars was the main achievement using founder parents in Ningxia bread wheat breeding. Gene flow was most frequent (2.47) when comparing modern cultivars with the founder parent Zhou 8425B for yield-related genes, meaning that founder parents, especially Zhou 8425B, have contributed greatly to the yield improvement of Ningxia wheat. Zhou 8425B is a founder parent fitting current breeding needs, with features of dwarfing, high yield and disease resistance, and more than 300 wheat varieties (lines) such as AK58 and Zhou 16 have been bred from this parent [65, 74, 75]. High yield is an ever-important objective of wheat breeding, and analysis of the breeding history of many crop species has revealed the presence and roles of founder parents [68]. Li et al. [76] found that Beijing 8, serving as a founder parent, contributed many loci in close proximity to the positions of known yield component genes that confer important traits in breeding. In addition, pedigree analysis has shown that inherited ancestor genome segments in the rice variety Huanghuazhan are extremely enriched in the grain yield category [73].

### Functional markers combining phenomics will advance Ningxia wheat breeding

Functional markers that have strong associations with relevant phenotypes are ideal for gene tagging, and allelic variants can be associated to functional genes in breeding [33, 77]. Liu et al. [33] documented 97 functional markers that detect 93 alleles at 30 loci in bread wheat. Rasheed et al. [39] converted gel-based functional markers to high-throughput KASP markers. In this study, we evaluated the molecular characterization and genetic distribution of Ningxia bread wheat breeding in terms of important genes related to adaptation, stress resistance, quality and yield by utilizing these KASP functional markers. However, an objective fact is that these in our research are only a few number of predicted genes relative to wheat whole genome. At present, many gene mapping studies (both QTL studies and GWAS) have identified genes controlling agronomic traits, and KASP markers have been produced [77]. Indeed, more than 150 KASP markers for almost 100 functional genes have been developed, and 72 have been validated in a bread wheat diversity panel [78]. With innovations of whole-genome assemblies, revolutionary advances in reference genome sequences for bread wheat ‘Chinese Spring’ [79] and its progenitors, *T. turgidum* spp. *dicoccoides* [80], *Aegilops tauschii* [81, 82] and *T. urartu* [83], have recently been achieved. These wheat genome data provide new opportunities to uncover genetic variation in traits of breeding interest and enable genome-based breeding to produce wheat cultivars. In the future, with an increasing number of important cloned genes, we will use more functional markers to genetically characterize Ningxia wheat.

With the development of sequencing technology, how to combine the data obtained from sequencing with practical breeding work has become the focus of breeders. Despite the functional markers for important cloned genes used in our study, a lack of relative phenotyping data has led to a poor understanding of the actual improvement obtained within the Ningxia breeding programme. Our capacity to collect useful high-quality phenotypic data lags behind the current capacity to generate genotyping data. However, 44 functional markers (genes) influencing grain yield, quality, adaptation and resistance phenotypes, such as plant height, vernalization, drought tolerance, leaf rust, grain hardness, grain width, and spikelet number per spike, have been developed, and collecting these phenotypic data is laborious, time consuming and costly due to the large number of bead wheat varieties. Luckily, with the rapid advancement of high-throughput plant phenotype measurement technology, plant phenomics has developed rapidly. High-throughput phenotyping platforms allow for recording data on traits such as plant development, architecture, growth, biomass, and photosynthesis for hundreds to thousands of plants in a single day [84], which will help to fill the gap of the lack of phenotypes in this study and benefit genomics-assisted breeding (GAB) for wheat improvement in Ningxia Province.

## Conclusions

In this study, we report a comprehensive functional gene assessment of modern improved wheat based on 44 important genes underlying grain yield, quality, adaptation and resistance in 207 cultivars and lines in Ningxia Province. Varieties introduced from other regions with rich genetic diversity and landraces with well-adapted genetic resources have been applied to improve modern cultivars. Founder parents, particularly Zhou 8425B, for yield-related genes have contributed greatly to breeding improvement of wheat in Ningxia Province. This work reports genetic characteristics at the gene level and advances improvement in selection for future wheat breeding in Ningxia Province.

## Supplementary Information

**Additional file 1: Table S1**. Detailed information of materials and their allelic variations of 44 genes used in this study. **Table S2**. Basic information including allelic variations and primer sequences for the 44 KASP assays. **Table S3**. Gene diversities and genetic differentiation (*Fst*) of 44 polymorphic genes between Ningxia and Others accessions. **Table S4**. Gene diversities and genetic differentiation (*Fst*) of 44 polymorphic genes between landraces and modern cultivars in Ningxia Province.

**Additional file 2: Fig. S1**. KASP genotyping at *Rht-D1*, *VP-1B* and *Glu-A1*. Red and blue dots show homozygous varieties; Green dots show heterozygous varieties; Black dots show negative control; X shows missing types.

**Additional file 3: Fig. S2**. Plot of Delta K against putative K ranging from 1 to 8.

**Additional file 4: Fig. S3**. Population structure and a neighbour-joining tree of 207 wheat accessions based on 44 genes. (A) Population structure of all accessions based on Structure from K = 2 to K = 3. (B) A neighbour-joining tree of 207 wheat accessions; Red asterisks represent six founder parents.

**Additional file 5: Fig. S4**. Genetic diversities on four types of genes between Ningxia and Others subgroups. (A) Genetic diversities on grain yield genes. (B) Genetic diversities on quality genes. (C) Genetic diversities on adaptation genes. (D) Genetic diversities on stress resistance genes.

**Additional file 6: Fig. S5**. Allele frequencies between Ningxia and Others subgroups at adaptation (A) and stress resistance (B) genes.

**Additional file 7: Fig. S6**. Genetic diversities on three types of genes between modern cultivars and landraces subgroups in Ningxia Province. (A) Genetic diversities on stress resistance genes. (B) Genetic diversities on quality genes. (C) Genetic diversities on grain yield genes.

**Additional file 8: Fig. S7**. Allele frequencies of adaptation genes between modern cultivars and landraces in Ningxia Province.

**Additional file 9: Fig. S8**. Allele frequencies of stress resistance genes between modern cultivars and landraces in Ningxia Province.

**Additional file 10: Fig. S9**. Allele frequencies of quality genes between modern cultivars and landraces in Ningxia Province.

**Additional file 11: Fig. S10**. Allele frequencies of grain yield genes between cultivars and landraces in Ningxia Province.

**Additional file 12: Fig. S11**. Distribution of allelic variations of grain yield genes in six founder parents.

**Additional file 13: Fig. S12**. Distribution of allelic variations of quality genes in six founder parents.

**Additional file 14: Fig. S13**. Distribution of allelic variations of stress resistance genes in six founder parents.

**Additional file 15: Fig. S14**. Distribution of allelic variations of adaptation genes in six founder parents.

## Data Availability

The datasets generated and analyzed during the current study are available from the corresponding author on reasonable requests.

## Abbreviations

*Fst*: F-statistics
HWM-GS: High-Molecular-Weight Glutenin Subunits
KASP: Kompetitive Allele Specific PCR
PCA: Principal Coordinate Analysis
MCMC: Markov Chain Monte Carlo
TKW: Thousand Kernel Weight
SNP: Single Nucleotide Polymorphism
LGC: Laboratory of the Government Chemist
HTBs: Huanghuazhan Traceable Blocks
GAB: genomics-assisted breeding
