# Supplementary figures and images for: Functional gene assessment of bread wheat: breeding implications in Ningxia Province
Source: BMC Plant Biol. 2021 Feb 18;21:103. doi: 10.1186/s12870-021-02870-5 (PMC7893757; doi:10.1186/s12870-021-02870-5)

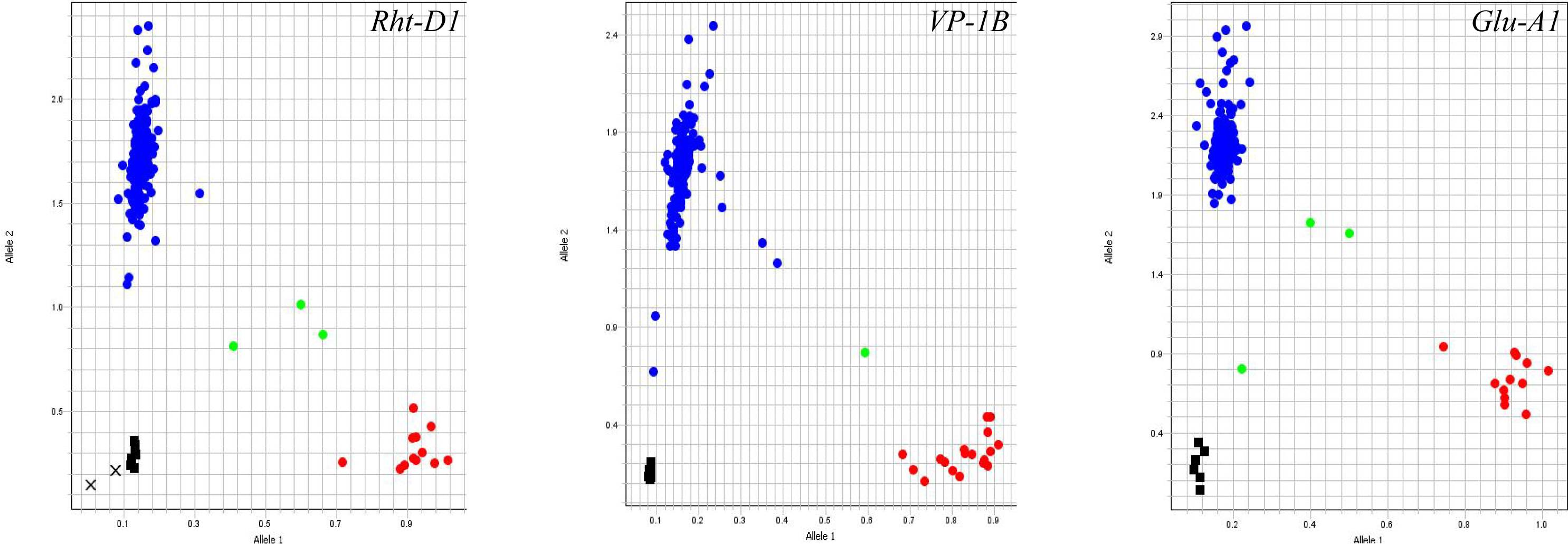

Supplement: Supplementary file 2 — Additional file 2: Fig. S1. KASP genotyping at Rht-D1, VP-1B and Glu-A1. Red and blue dots show homozygous varieties; Green dots show heterozygous varieties; Black dots show negative control; X shows missing types. [file 12870_2021_2870_MOESM2_ESM.tif]

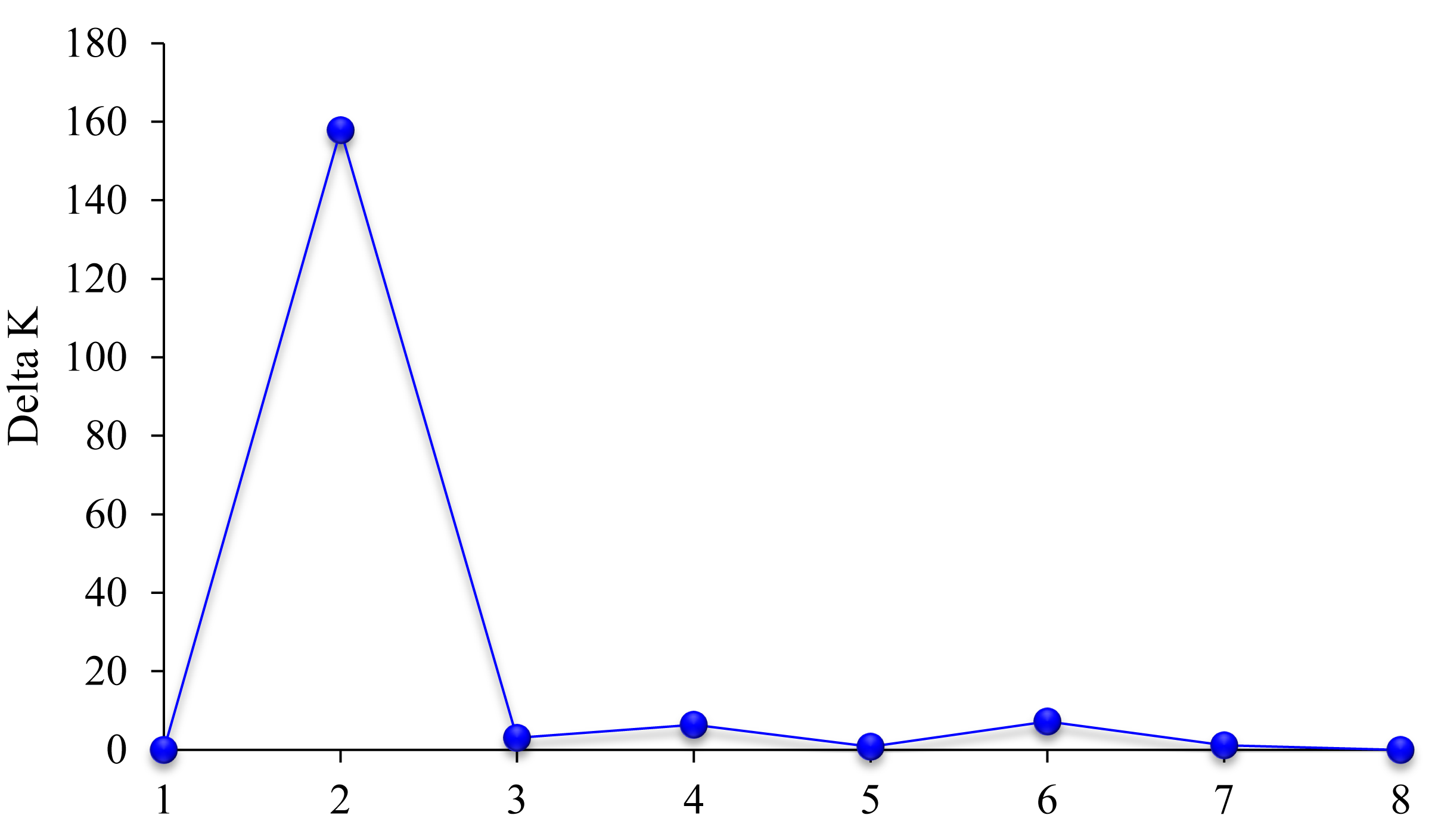

Supplement: Supplementary file 3 — Additional file 3: Fig. S2. Plot of Delta K against putative K ranging from 1 to 8. [file 12870_2021_2870_MOESM3_ESM.tif]

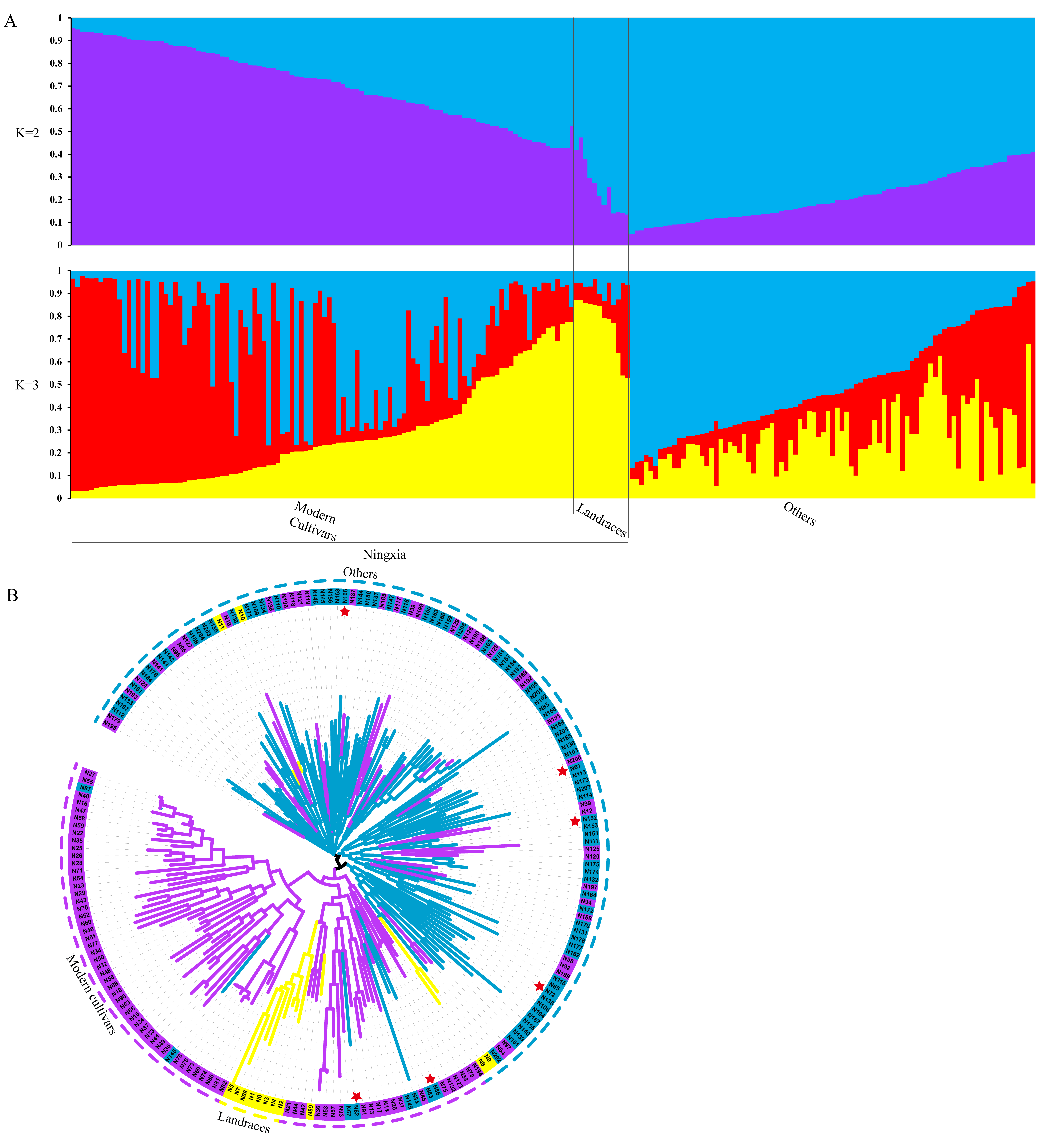

Supplement: Supplementary file 4 — Additional file 4: Fig. S3. Population structure and a neighbour-joining tree of 207 wheat accessions based on 44 genes. (A) Population structure of all accessions based on Structure from K = 2 to K = 3. (B) A neighbour-joining tree of 207 wheat accessions; Red asterisks represent six founder parents. [file 12870_2021_2870_MOESM4_ESM.tif]

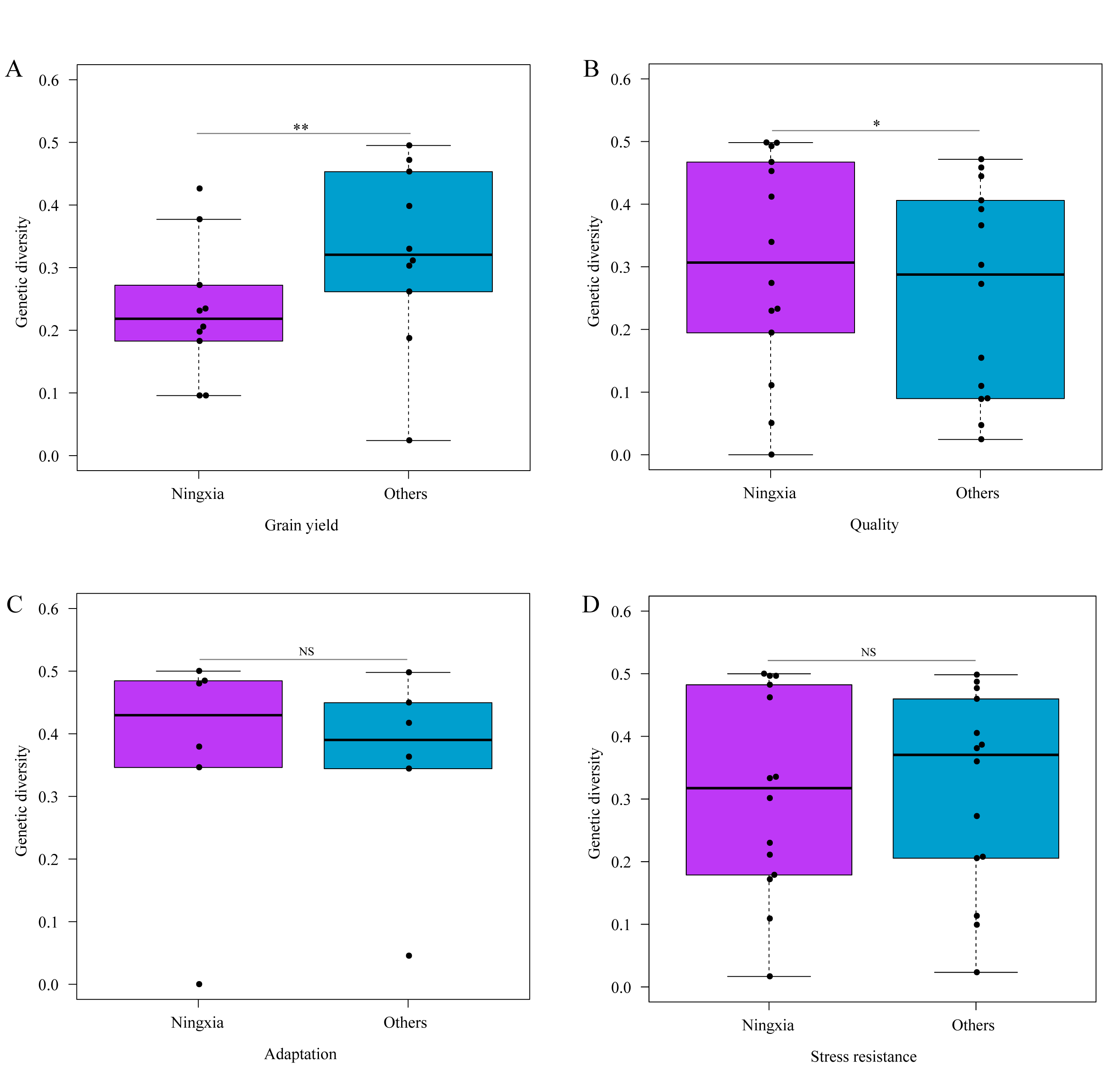

Supplement: Supplementary file 5 — Additional file 5: Fig. S4. Genetic diversities on four types of genes between Ningxia and Others subgroups. (A) Genetic diversities on grain yield genes. (B) Genetic diversities on quality genes. (C) Genetic diversities on adaptation genes. (D) Genetic diversities on stress resistance genes. [file 12870_2021_2870_MOESM5_ESM.tif]

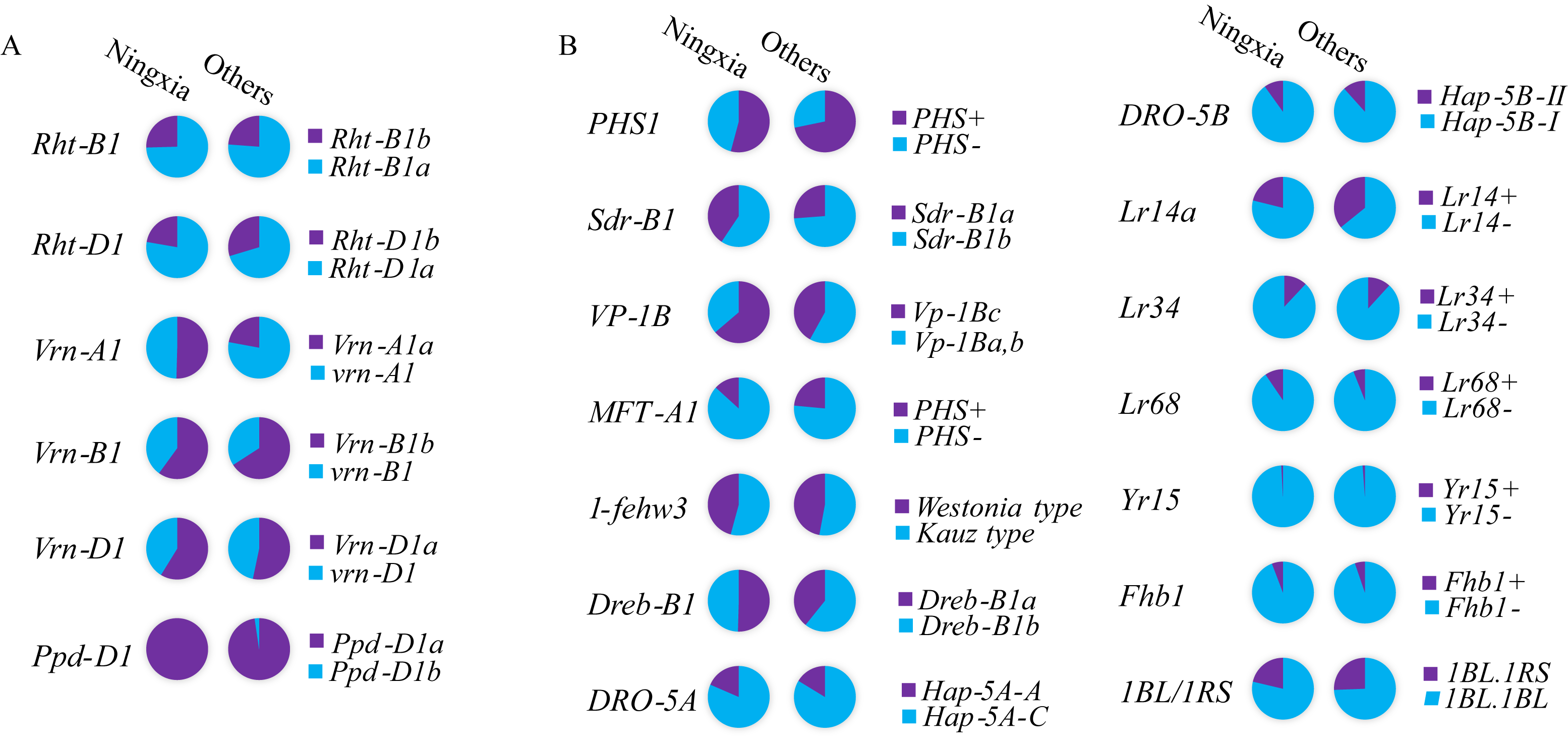

Supplement: Supplementary file 6 — Additional file 6: Fig. S5. Allele frequencies between Ningxia and Others subgroups at adaptation (A) and stress resistance (B) genes. [file 12870_2021_2870_MOESM6_ESM.tif]

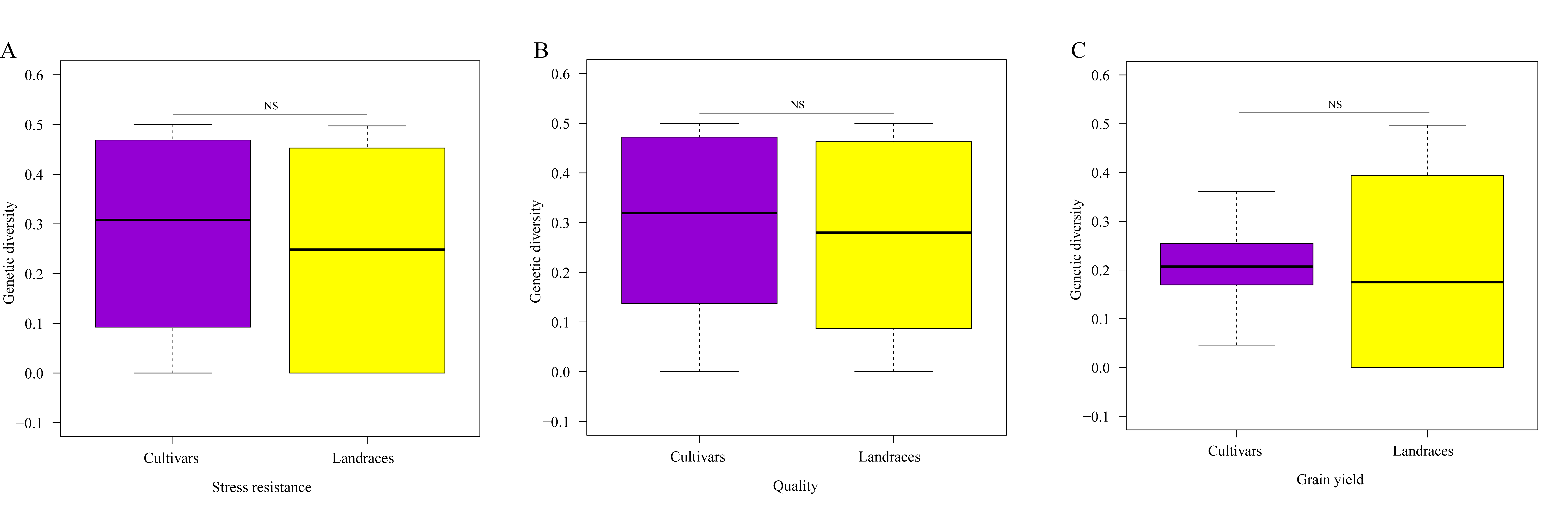

Supplement: Supplementary file 7 — Additional file 7: Fig. S6. Genetic diversities on three types of genes between modern cultivars and landraces subgroups in Ningxia Province. (A) Genetic diversities on stress resistance genes. (B) Genetic diversities on quality genes. (C) Genetic diversities on grain yield genes. [file 12870_2021_2870_MOESM7_ESM.tif]

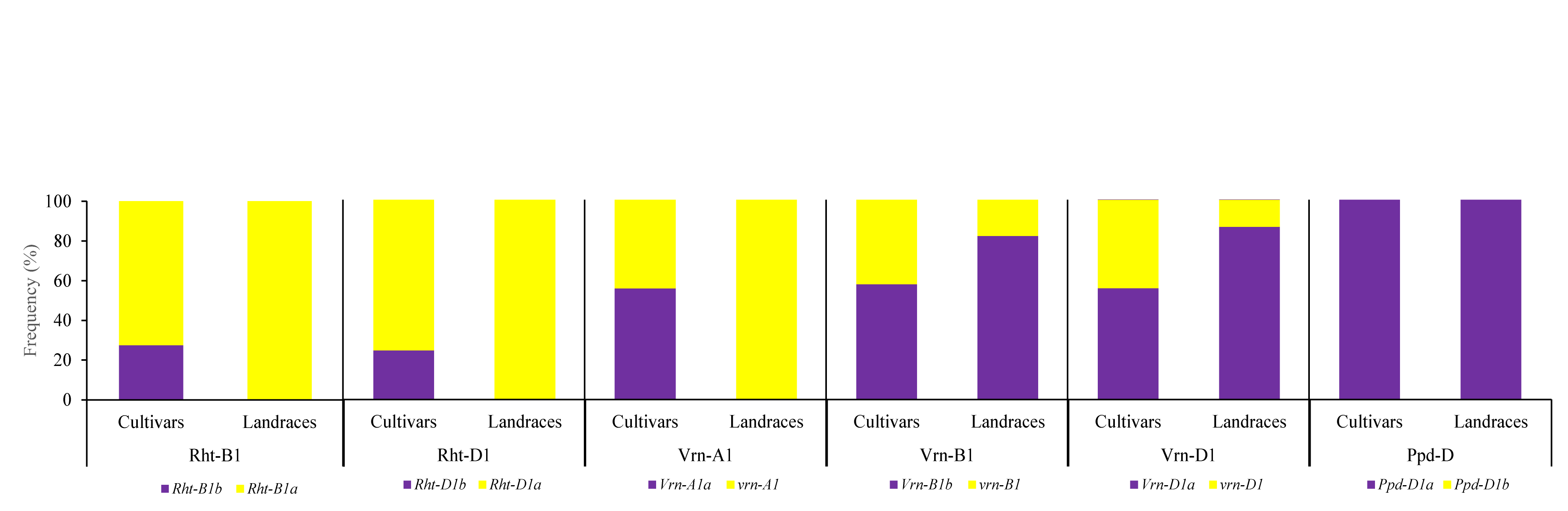

Supplement: Supplementary file 8 — Additional file 8: Fig. S7. Allele frequencies of adaptation genes between modern cultivars and landraces in Ningxia Province. [file 12870_2021_2870_MOESM8_ESM.tif]

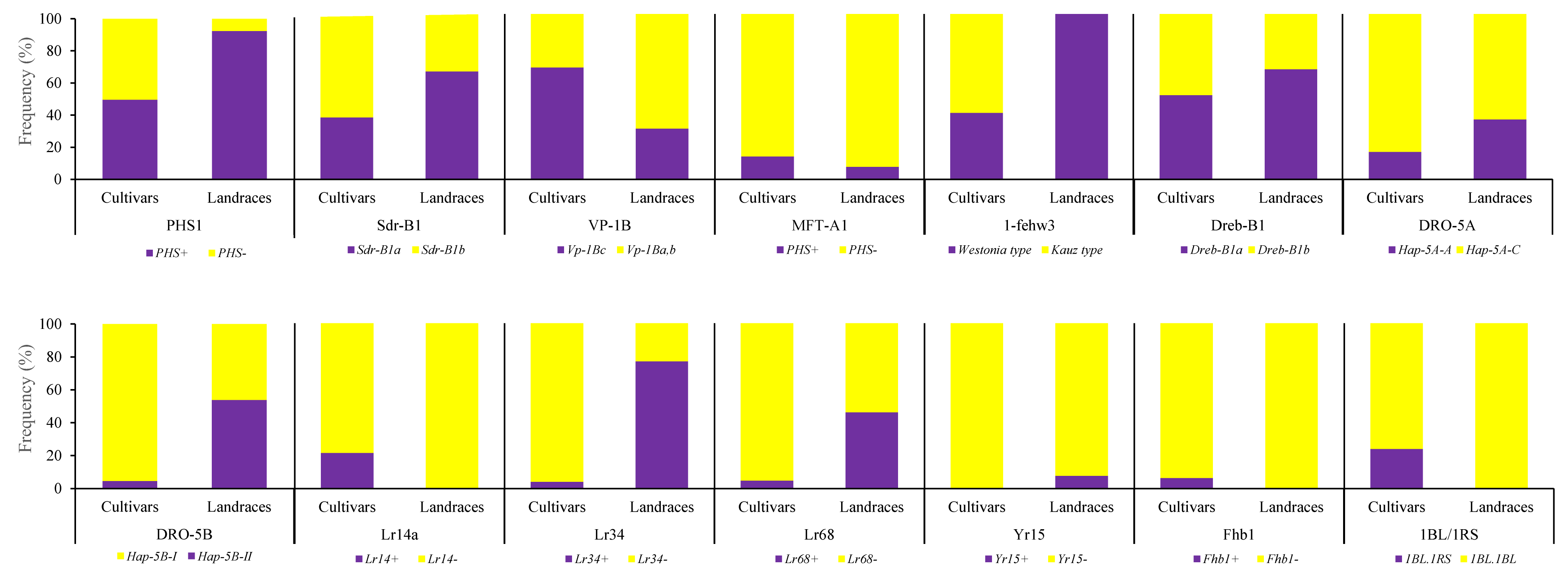

Supplement: Supplementary file 9 — Additional file 9: Fig. S8. Allele frequencies of stress resistance genes between modern cultivars and landraces in Ningxia Province. [file 12870_2021_2870_MOESM9_ESM.tif]

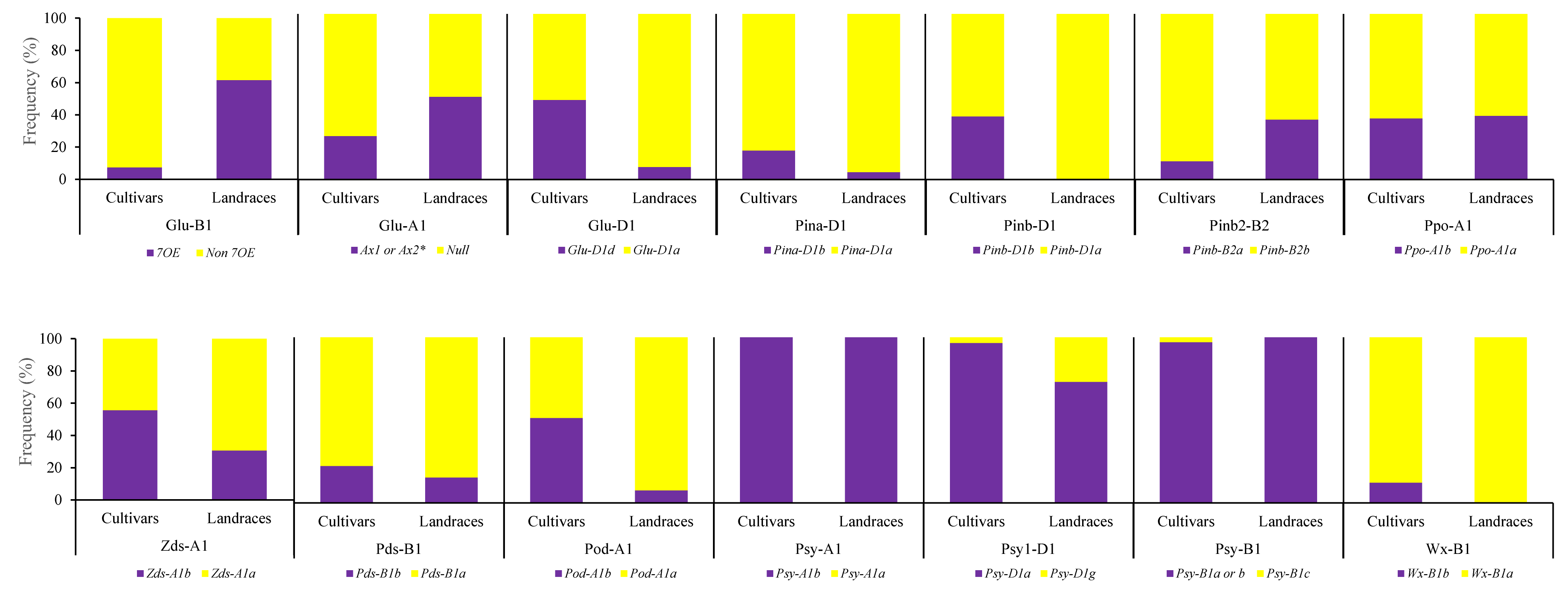

Supplement: Supplementary file 10 — Additional file 10: Fig. S9. Allele frequencies of quality genes between modern cultivars and landraces in Ningxia Province. [file 12870_2021_2870_MOESM10_ESM.tif]

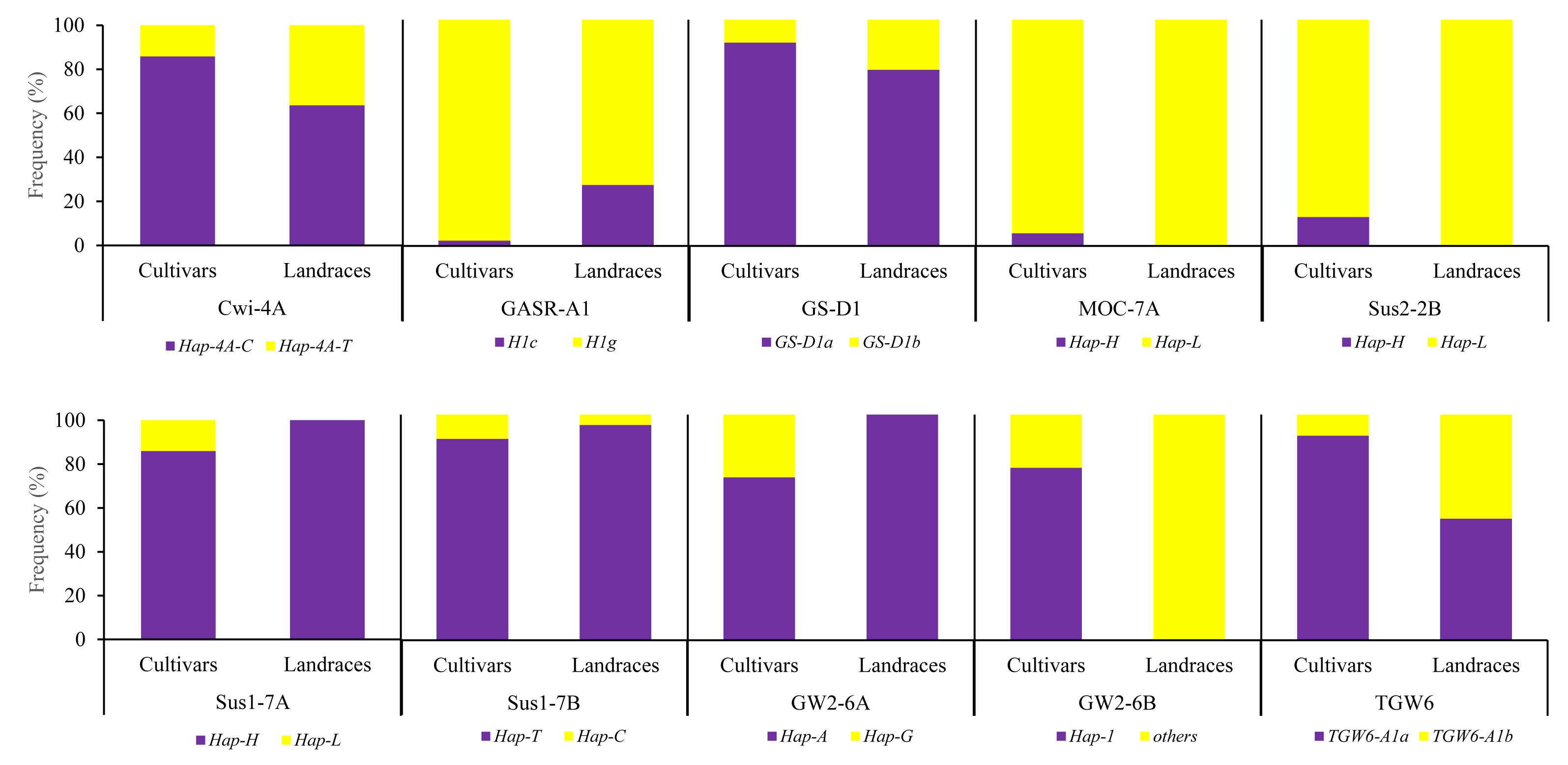

Supplement: Supplementary file 11 — Additional file 11: Fig. S10. Allele frequencies of grain yield genes between cultivars and landraces in Ningxia Province. [file 12870_2021_2870_MOESM11_ESM.tif]

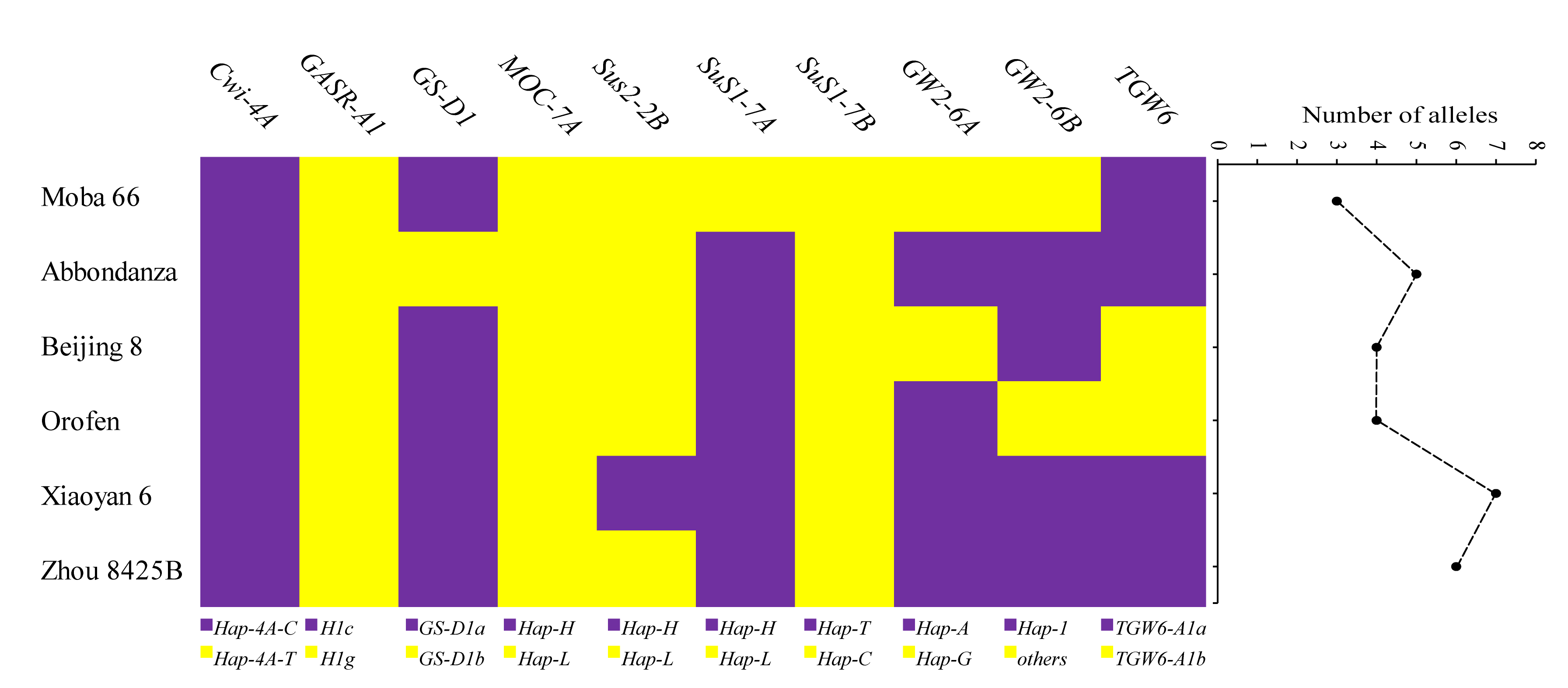

Supplement: Supplementary file 12 — Additional file 12: Fig. S11. Distribution of allelic variations of grain yield genes in six founder parents. [file 12870_2021_2870_MOESM12_ESM.tif]

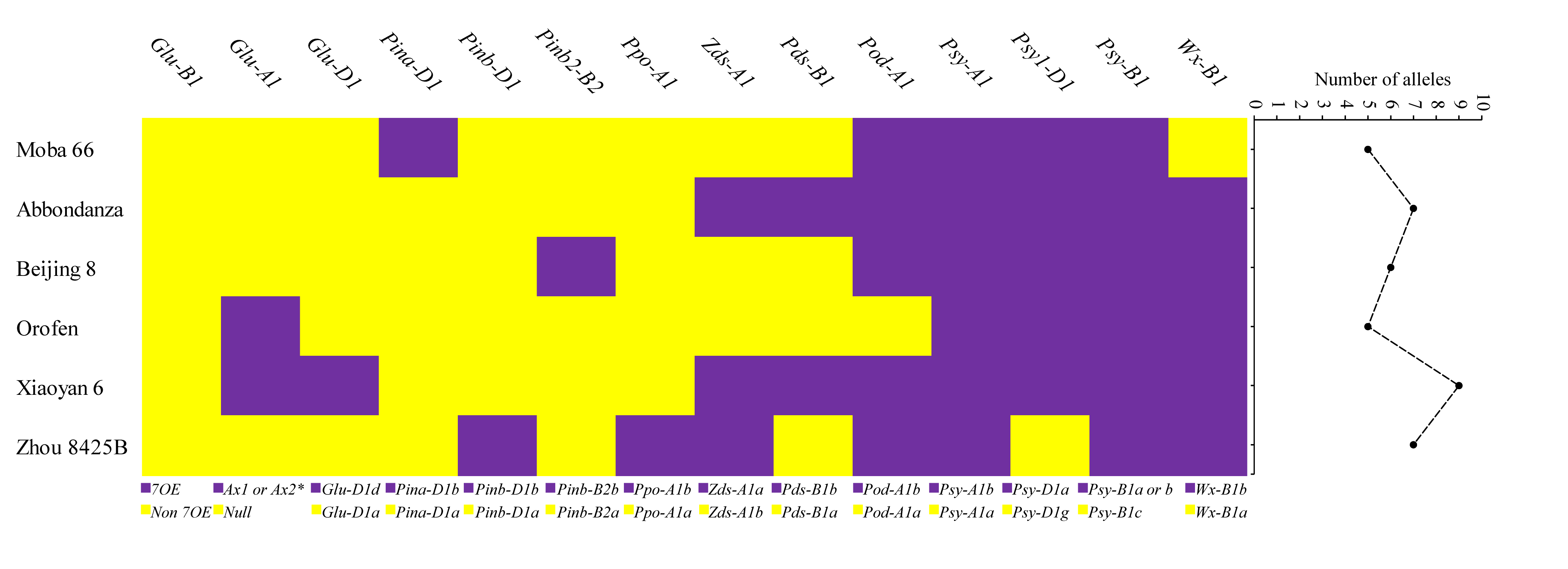

Supplement: Supplementary file 13 — Additional file 13: Fig. S12. Distribution of allelic variations of quality genes in six founder parents. [file 12870_2021_2870_MOESM13_ESM.tif]

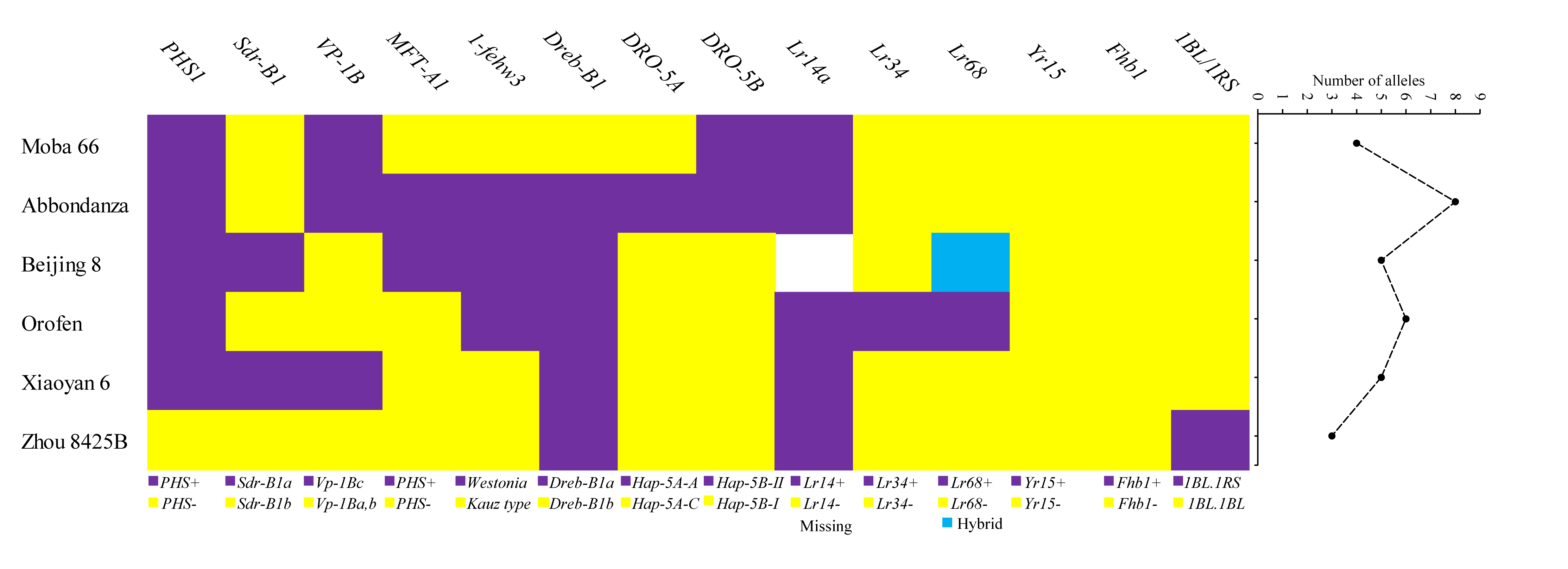

Supplement: Supplementary file 14 — Additional file 14: Fig. S13. Distribution of allelic variations of stress resistance genes in six founder parents. [file 12870_2021_2870_MOESM14_ESM.tif]

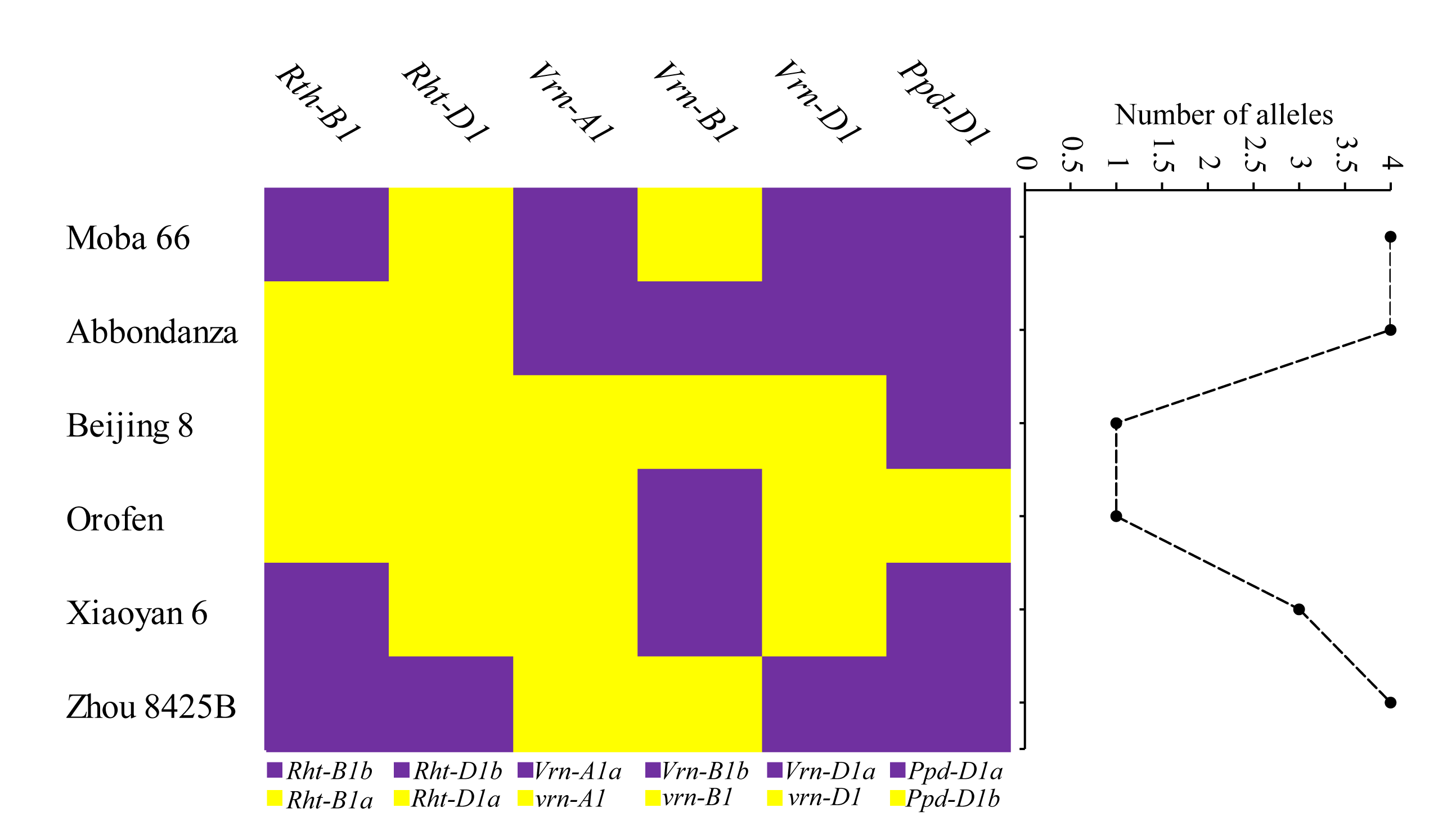

Supplement: Supplementary file 15 — Additional file 15: Fig. S14. Distribution of allelic variations of adaptation genes in six founder parents. [file 12870_2021_2870_MOESM15_ESM.tif]
